# Hippocampal PACAP signaling activation triggers a rapid antidepressant response

**DOI:** 10.1186/s40779-024-00548-1

**Published:** 2024-07-23

**Authors:** Hai-Lou Zhang, Yan Sun, Zhang-Jie Wu, Ying Yin, Rui-Yi Liu, Ji-Chun Zhang, Zhang-Jin Zhang, Suk-Yu Yau, Hao-Xin Wu, Ti-Fei Yuan, Li Zhang, Miroslav Adzic, Gang Chen

**Affiliations:** 1 Interdisciplinary Institute for Personalized Medicine in Brain Disorders, Jinan University, Guangzhou, 510632 China; 2 Departments of Psychiatry & Clinical and Translational Institute of Psychiatric Disorders, First Affiliated Hospital of Jinan University, Guangzhou, 510632 China; 3 The Guangdong-Hongkong-Macau Joint Laboratory of Traditional Chinese Medicine Regulation of Brain-Periphery Homeostasis and Comprehensive Health, Guangzhou, 510632 China; 4 Key Laboratory of Integrative Biomedicine for Brain Diseases, School of Chinese Medicine, Nanjing University of Chinese Medicine, Nanjing, 210023 China; 5 School of Medicine, Jinan University, Guangzhou, 510632 China; 6 School of Chinese Medicine, LKS Faculty of Medicine, the University of Hong Kong, Hong Kong, 999077 China; 7 Department of Rehabilitation Sciences, Hong Kong Polytechnic University, Hong Kong, 999077 China; 8 Shanghai Mental Health Center, Shanghai, 200030 China; 9 Key Laboratory of Central CNS Regeneration (Ministry of Education), Guangdong-Hong Kong-Macau Institute of CNS Regeneration, Jinan University, Guangzhou, 510632 China; 10 “Vinča” Institute of Nuclear Sciences, Laboratory of Molecular Biology and Endocrinology 090, University of Belgrade, 11001 Belgrade, Serbia

**Keywords:** Antidepressant response, Pituitary adenylate cyclase-activating polypeptide (PACAP), Ketamine, Optogenetic, Novelty suppressed feeding (NSF)

## Abstract

**Background:**

The development of ketamine-like rapid antidepressants holds promise for enhancing the therapeutic efficacy of depression, but the underlying cellular and molecular mechanisms remain unclear. Implicated in depression regulation, the neuropeptide pituitary adenylate cyclase-activating polypeptide (PACAP) is investigated here to examine its role in mediating the rapid antidepressant response.

**Methods:**

The onset of antidepressant response was assessed through depression-related behavioral paradigms. The signaling mechanism of PACAP in the hippocampal dentate gyrus (DG) was evaluated by utilizing site-directed gene knockdown, pharmacological interventions, or optogenetic manipulations. Overall, 446 mice were used for behavioral and molecular signaling testing. Mice were divided into control or experimental groups randomly in each experiment, and the experimental manipulations included: chronic paroxetine treatments (4, 9, 14 d) or a single treatment of ketamine; social defeat or lipopolysaccharides-injection induced depression models; different doses of PACAP (0.4, 2, 4 ng/site; microinjected into the hippocampal DG); pharmacological intra-DG interventions (CALM and PACAP6-38); intra-DG viral-mediated PACAP RNAi; and opotogenetics using channelrhodopsins 2 (ChR2) or endoplasmic natronomonas halorhodopsine 3.0 (eNpHR3.0). Behavioral paradigms included novelty suppressed feeding test, tail suspension test, forced swimming test, and sucrose preference test. Western blotting, ELISA, or quantitative real-time PCR (RT-PCR) analysis were used to detect the expressions of proteins/peptides or genes in the hippocampus.

**Results:**

Chronic administration of the slow-onset antidepressant paroxetine resulted in an increase in hippocampal PACAP expression, and intra-DG blockade of PACAP attenuated the onset of the antidepressant response. The levels of hippocampal PACAP expression were reduced in both two distinct depression animal models and intra-DG knockdown of *PACAP *induced depression-like behaviors. Conversely, a single infusion of PACAP into the DG region produced a rapid and sustained antidepressant response in both normal and chronically stressed mice. Optogenetic intra-DG excitation of PACAP-expressing neurons instantly elicited antidepressant responses, while optogenetic inhibition induced depression-like behaviors. The longer optogenetic excitation/inhibition elicited the more sustained antidepressant/depression-like responses. Intra-DG PACAP infusion immediately facilitated the signaling for rapid antidepressant response by inhibiting calcium/calmodulin-dependent protein kinase II (CaMKII)-eukaryotic elongation factor 2 (eEF2) and activating the mammalian target of rapamycin (mTOR). Pre-activation of CaMKII signaling within the DG blunted PACAP-induced rapid antidepressant response as well as eEF2-mTOR-brain-derived neurotrophic factor (BDNF) signaling. Finally, acute ketamine treatment upregulated hippocampal PACAP expression, whereas intra-DG blockade of PACAP signaling attenuated ketamine’s rapid antidepressant response.

**Conclusions:**

Activation of hippocampal PACAP signaling induces a rapid antidepressant response through the regulation of CaMKII inhibition-governed eEF2-mTOR-BDNF signaling.

**Supplementary Information:**

The online version contains supplementary material available at 10.1186/s40779-024-00548-1.

## Background

Major depressive disorder (MDD) is a debilitating mental illness that affects millions of individuals worldwide, resulting in significant health and socioeconomic consequences [1, 2]. Conventional antidepressant medications, typically selective serotonin reuptake inhibitors (SSRIs), are associated with notable limitations including delayed onset of efficacy and non-responsive subpopulations [3, 4]. In contrast, recent evidence has demonstrated that a single administration of ketamine induces a rapid onset of antidepressant response within hours, which persists for several days in both MDD patients and animal models of depression [5, 6]. Given the heightened risk of suicide in MDD patients, the fast-acting antidepressant properties akin to ketamine offer promise for enhancing MDD treatment; however, further elucidation of the underlying neuromolecular mechanisms is warranted.

The findings of rapid antidepressant response provide support for the neuroplasticity hypothesis regarding depression and antidepressant response: in the hippocampus and associated neural circuits, impaired neural plasticity and underlying signaling contribute to depression, while enhanced neural plasticity governs the onset of rapid or delayed antidepressant response [7]. The crucial molecular machinery involved in post-transcriptional regulation of protein synthesis has been revealed for the rapid antidepressant response, including inhibition of eukaryotic elongation factor 2 (eEF2) signaling or activation of mammalian target of rapamycin (mTOR) signaling [8, 9]. Furthermore, synaptic plasticity-related proteins such as brain-derived neurotrophic factor (BDNF) and postsynaptic density protein-95 (PSD95) were rapidly upregulated [9–11]. Additionally, there is an association between rapid antidepressant inhibition of eEF2 or activation of mTOR with calcium/calmodulin-dependent protein kinase II (CaMKII), although the causal link between CaMKII and mTOR/eEF2 remains unclear [12, 13]. Moreover, the specific neuromolecular substrates modulating these signaling are largely unknown.

Human genetic variants of pituitary adenylate cyclase-activating polypeptide (PACAP) or its receptors have been associated with psychiatric disorders, including post-traumatic stress disorder, anxiety, and depression [14, 15]. PACAP is one of the most intensively investigated neuropeptides that functions as a neurohormone, neurotransmitter, and neurotrophic mediator [16]. As a potent activator of cyclic adenosine monophosphate (cAMP), PACAP induces various downstream signaling cascades that ultimately enhance neural plasticity in the brain [17]. The classic downstream signaling pathway of PACAP involves protein kinase A (PKA)-cAMP-response element binding protein (CREB), which upregulates gene translations. Recent studies also indicate that PACAP activates mTOR [13, 18, 19]. In animal studies, some *PACAP* knockout mice showed attenuated depression-like responses to stress, but the role of PACAP in the rapid antidepressant effect remains elusive [16, 20–22]. It has been demonstrated that a single dose of PACAP robustly facilitates both immediate and long-term synaptic plasticity [23], suggesting that its ability to enhance neural plasticity may confer a strong antidepressant capacity. Administration of certain SSRIs including paroxetine or some natural compounds has been found to increase hippocampal expression of PACAP and elicit antidepressant responses [13, 24]. Furthermore, higher levels of hippocampal PACAP expression after several day treatments with paroxetine were associated with better antidepressant response, suggesting the involvement of hippocampal PACAP signaling in the onset of an antidepressant response [25].

The dentate gyrus (DG) hilus in the hippocampus is prominently populated by PACAP-expressing neurons, and the receptors are intensively expressed in the granule cells and their projection neurons in cornu ammonis area 3 (CA3). However, the function of these neurons remains largely unknown [16, 22]. This study aimed to reveal that activation of PACAP^DG^ signaling or PACAP^DG^ expressing neurons triggers the onset of antidepressant response through enhancement of neuroplasticity signaling induced by chronic paroxetine or acute ketamine.

## Methods

### Animals

A total of 446 mice were used in the study. All animal care and experimental procedures conformed to the Guide for the Care and Use of Laboratory Animals and were approved by the Institutional Animal Care and Use Committee at Nanjing University of Chinese Medicine (201904A003) and Jinan University (20210303-43). Welfare-related assessments, measurements, and interventions were carried out before, during, and after the experiment. Male and female Kunming (RRID: MGI: 5651867; *n* = 294, half male and half female) or 129S1/SvImJ (RRID: MGI: 5658424, *n* = 104) strain mice were purchased from Shanghai Sippr-BK Laboratory Animal Co., Ltd. (SCXK [Hu] 2018-0006) in China, and Adcyap1-2A-Cre knock-in mice were purchased from Jackson Laboratory (Stock#030155; *n* = 48). Animals aged 6 – 8 weeks old, 20 – 25 g, were habituated to animal facilities for 1 week before the experiment. They were kept at (21 ± 1) ℃ with (50 ± 10)% humidity in a 12 h light/dark cycle with ad libitum food and water throughout the experiment and group-housed (5 mice per cage, cage size: 318 mm × 202 mm × 135 mm) with corncobs bedding under strict hygienic conventional conditions.

### Protocol of depression model and behavioral tests

The previously reported procedures of depression-related behavioral tests of novelty suppressed feeding (NSF), tail suspension test (TST), forced swimming test (FST), open field test (OFT), sucrose preference test (SPT), and the social defeat (SD) depression model were followed with minor modifications [26], with details in Additional file 1: Materials and methods. In the lipopolysaccharide (LPS)-induced depression model, the mice were administrated with a single dose of LPS (0.5 mg/kg, i.p.; HY-D1056, Sigma, USA) per day for 4 consecutive days [27].

### Drugs

PACAP 1-38 (0.4, 2.0, 4.0 ng/site; PACAP signaling agonist, 137061-48-4, MedChemExpress, USA), PACAP 6-38 (0.04 ng/site; PACAP signaling antagonist, HY-P0220, MedChemExpress, USA) and CALM (40 ng/site; CaMKII synthetic substrate, C911, Novaprotein, China) were dissolved in 1% bovine serum albumin (Roche, USA), administrated intrahippocampally in a volume of 0.2 μl. Ketamine (30 mg/kg; KH140303, Gutian Pharmaceuticals, China) dissolved in saline, was administered intraperitoneally and the dose of ketamine was chosen according to previous studies [17]. LPS was dissolved in DMSO (1%) of saline solution for improvement of its absorption [27].

### Western blotting, ELISA, immunofluorescence, quantitative real-time PCR analysis

Mice were immediately euthanized by cervical dislocation at the designated time points. Within 2 min, tissues were dissected and collected on ice and frozen quickly. Western blotting, ELISA, immunofluorescence, and quantitative real-time PCR analysis are described in detail in Additional file 1: Materials and methods.

### Microinfusion and intrahippocampal RNAi

Mice were anesthetized using pentobarbital sodium (45 mg/kg, i.p.; 57-33-0, Sigma, USA) and placed on a stereotactic apparatus for surgery. During the operation, all the mice were given continuous oxygen by ventilators for spontaneous breathing. The coordinates for the hippocampus DG were: ML ± 0.2 cm; AP -0.21 cm from Bregma; DV -0.24 cm from dura. In general, no antibiotics or analgesic was used. Animals recovered for 1 week or 3 weeks before micro-infusion or optogenetic experimentation, respectively. Histological verification was carried out to only include the data from the mice injected into DG for further analysis. The procedure for surgery and tests for micro-infusion or viral transfection are described in detail in Additional file 1: Materials and methods.

### Optogenetic experiments

Bilateral DG of the Adcyap1-2A-Cre knock-in mice were injected based on the above-mentioned coordinates with AAV-Ef1a-DIO-channelrhodopsins 2 (ChR2)-mCherry or the control AAV-Ef1a-DIO-mCherry. Pulsed optogenetic activation was set as follows: LED, blue light, 473 nm, 10 Hz, 10 mW/mm^2^. The same procedure was followed for opto-inhibition of DG PACAP activity, except that AAV-Ef1a-DIO-endoplasmic natronomonas halorhodopsine 3.0 (eNpHR3.0)-mCherry or AAV-Ef1a-DIO-mCherry (OBiO, Shanghai, China) was used, and pulsed optogenetic inhibition was set as follows: LED, yellow light, 590 nm, 10 Hz, 10 mW/mm^2^. The electrophysiology recording was performed to verify the effects of the optogenetic manipulation (Additional file 1: Materials and methods). Histological verification was carried out to only include the data from the mice injected into DG for further analysis.

### Statistical analysis

The details on the blind and random procedure were described in the Additional file 1: Materials and methods. Data were expressed as means ± SEM. Unless indicated, statistical analyses were made by student *t*-test, two-tailed for two samples. One-way or two-way ANOVA with Tukey’s comparison test for independent measurement was used for Pearson’s correlation coefficients. Differences were considered to be significant at* P* < 0.05.

## Results

### Requirement of upregulation in hippocampal PACAP expression for the onset of the antidepressant response following paroxetine treatment

**Fig. 1:**
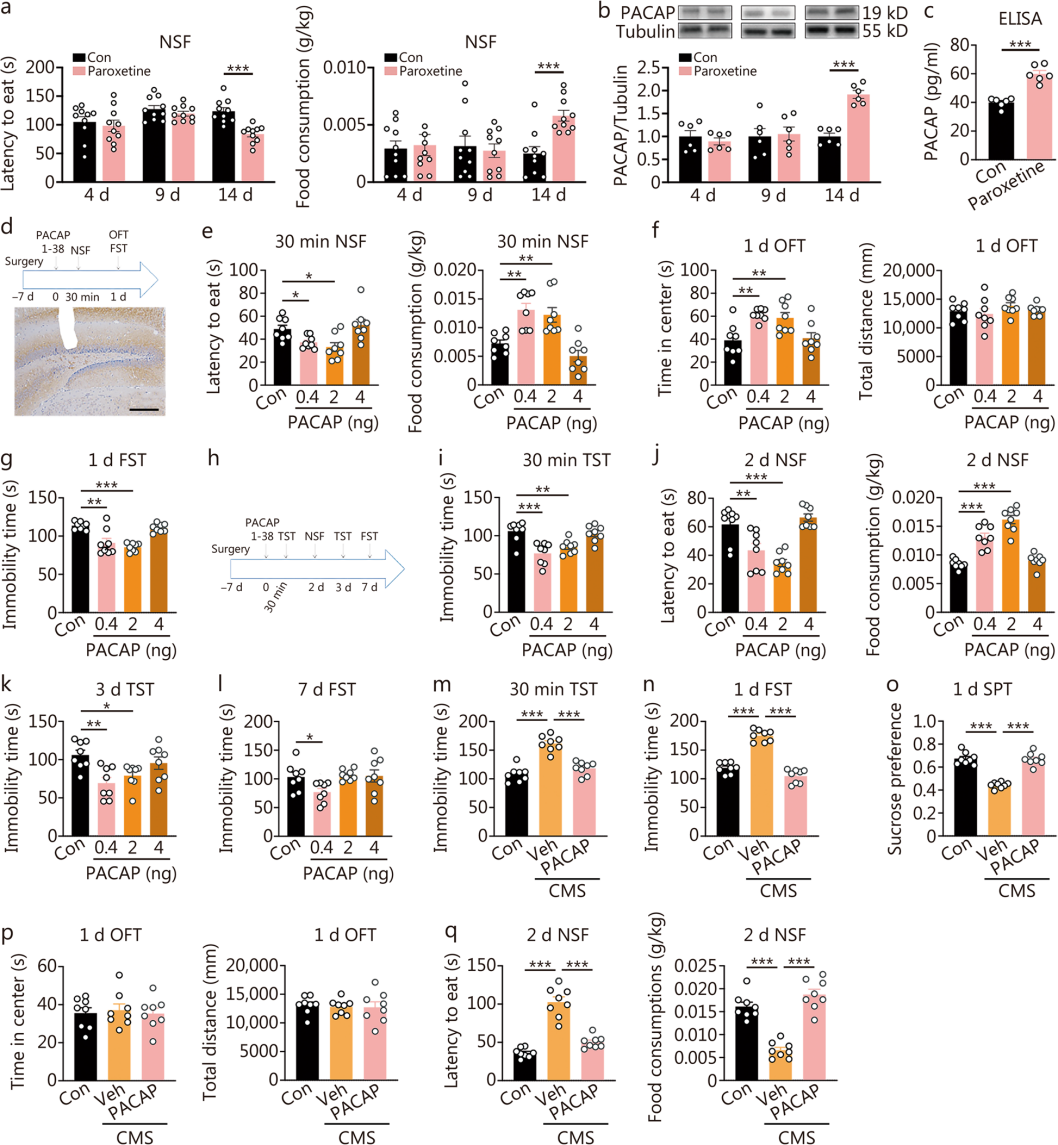
Effects of augment or attenuation of hippocampal pituitary adenylate cyclase-activating polypeptide (PACAP) signaling on the onset of antidepressant response or depression-like behaviors. Requirement of hippocampal PACAP expression upregulation for the onset of the antidepressant response following paroxetine treatment. **a** Latency to eat and food consumption in novelty-suppressed feeding (NSF) at 4, 9, 14 d by paroxetine treatment (*n* = 10). **b** The expression of PACAP in hippocampus 4, 9, 14 d by paroxetine treatment by Western blotting analysis. **c** The expression of PACAP in the hippocampus 14 d by paroxetine treatment by ELISA analysis (*n* = 6). *t*-test, ^***^P < 0.001. Intra-dentate gyrus (DG) PACAP infusion elicited a rapid and long-lasting antidepressant response. **d** The procedure of experimental design, drug treatment and behavioral test (upper), and the tract of the cannulation for intra-DG infusion of PACAP (below, scale bar = 100 μm). **e** Latency to eat and food consumption in NSF at 30 min post-PACAP [*F* (3, 28) = 5.940, *P* = 0.0029; *F* (3, 28) = 14.16, *P* < 0.0001]. **f** Time in the center and total distance traveling in open filed test (OFT) at 1 d post-PACAP [*F* (3, 28) = 7.349, *P* = 0.0009; *F* (3, 28) = 0.8864, *P* = 0.4602]. **g** Immobility time in forced swimming test (FST) at 1 d post-PACAP [*F* (3, 28) = 16.99, *P* < 0.0001]. **h** The experiment was designed for tests of long-term antidepressant effects by different doses of PACAP. **i** Immobility time in tail suspension test (TST) at 30 min post-PACAP [*F* (3, 28) = 10.82, *P* < 0.0001]. **j** Latency to eat and food consumption in NSF at 2 d post-PACAP [*F* (3, 28) = 16.35, *P* < 0.0001; *F* (3, 28) = 41.92, *P* < 0.0001]. **k** Immobility time in TST at 3 d post-PACAP [*F* (3, 28) = 5.658, *P* = 0.0037]. **l** Immobility time in FST at 7 d post-PACAP [*F* (3, 28) = 3.415, *P* = 0.0309] (*n* = 8). One-way ANOVA, ^*^P < 0.05, ^**^P < 0.01, ^***^P < 0.001. The immediate and lasting antidepressant activity with the infusion of 0.4 ng/site of hippocampal PACAP in mice subjected to chronic mild stress (CMS). **m** Behavioral comparisons across different groups, including immobility time in the TST at 30 min [*F* (2, 21) = 39.74, *P* < 0.0001]. **n** Post-PACAP-infusion, immobility time in the FST [*F* (2, 21) = 96.85, *P* < 0.0001]. **o** Sucrose preference in the SPT [*F* (2, 21) = 63.94, *P* < 0.0001]. **p** Time in the center and total distance in OFT [*F* (2, 21) = 0.1119, *P* = 0.8947; *F* (2, 21) = 0.08789,* P* = 0.9162] at day 1 post-PACAP [*F* (2, 21) = 0.1119, *P* = 0.8947; *F* (2, 21) = 0.08789,* P* = 0.9162]. **q** Latency to eat and food consumption in NSF [*F* (2, 21) = 61.25, *P* < 0.0001; *F* (2, 21) = 45.74,* P* < 0.0001] at day 2 post-PACAP [*F* (2, 21) = 61.25, *P* < 0.0001; *F* (2, 21) = 45.74,* P* < 0.0001] (*n* = 7 – 8). One-way ANOVA, ^***^P < 0.001

To investigate the correlation between the duration of paroxetine treatment with PACAP expression, as well as its impact on the onset of antidepressant response, we conducted a time-course study examining both PACAP expression and behavioral effects following different durations of paroxetine administration (Fig. 1a-c). Paroxetine was administrated for 4, 9, and 14 d respectively. Neither the 4-day nor 9-day administration of paroxetine resulted in improved performance in the NSF test, a behavioral paradigm used to detect signs of fast-onset antidepressant response [6], nor were there any changes observed in PACAP expression levels. However, after 14 d, NSF performance showed improvement (Fig. 1a), accompanied by an upregulation in hippocampal PACAP expression confirmed through Western blotting and ELISA analysis (Fig. 1b, c).

To further investigate whether enhancing PACAP signaling in the hippocampal DG can induce a rapid antidepressant response and determine its duration, we conducted a dose-effect and time-effect relationship study by micro-infusion of PACAP 1-38 (0.4, 2, 4 ng/site) bilaterally into the DG (Fig. 1d-l). After 30 min of PACAP infusion at both low and medium doses, we observed a reduction in latency to eat and an increase in food consumption in NSF (Fig. 1e). Additionally, there was a significant increase in time spent in the center during the OFT with both low and medium doses, while the total distance traveled remained unchanged after 1 d post-PACAP infusion (Fig. 1f). Moreover, immobility time during the FST was significantly reduced by both low and medium doses after 1 d post-PACAP infusion (Fig. 1g). We also found that immobility time during TST was significantly reduced by both low and medium doses after 30 min post-PACAP infusion (Fig. 1i). There was a significant improvement in NSF performance at 2 d post-PACAP infusion (Fig. 1j), as well as there was a reduction in immobility time during TST with both low and medium doses at 3 d post-PACAP infusion (Fig. 1k). Only the mice administrated with 0.4 ng/site still showed antidepressant response at 7 d post-PACAP-infusion as demonstrated by a significant reduction of immobility time during FST (Fig. 1l).

The rapid and enduring antidepressant effects of a single infusion of PACAP in the hippocampus were further confirmed in mice exposed to chronic mild stress (Fig. 1m-q). The administration of PACAP immediately reversed depression-like behaviors induced by chronic mild stress, as evidenced by a reduction in immobility time in the TST at 30 min post-infusion (Fig. 1m), a decrease in immobility time in the FST at 1 d post-infusion (Fig. 1n), an increase in sucrose preference in the SPT at 1 d post-infusion (Fig. 1o), and a decrease in the latency to eat and an increase in food consumption in the NSF at 2 d post-infusion (Fig. 1q). There were no changes observed for time spent in the center or total distance traveled within the OFT due to stress or PACAP infusion (Fig. 1p).

### SD stress or LPS injections attenuated the hippocampal PACAP levels and knockdown of DG PACAP-induced depression-like behaviors

**Fig. 2:**
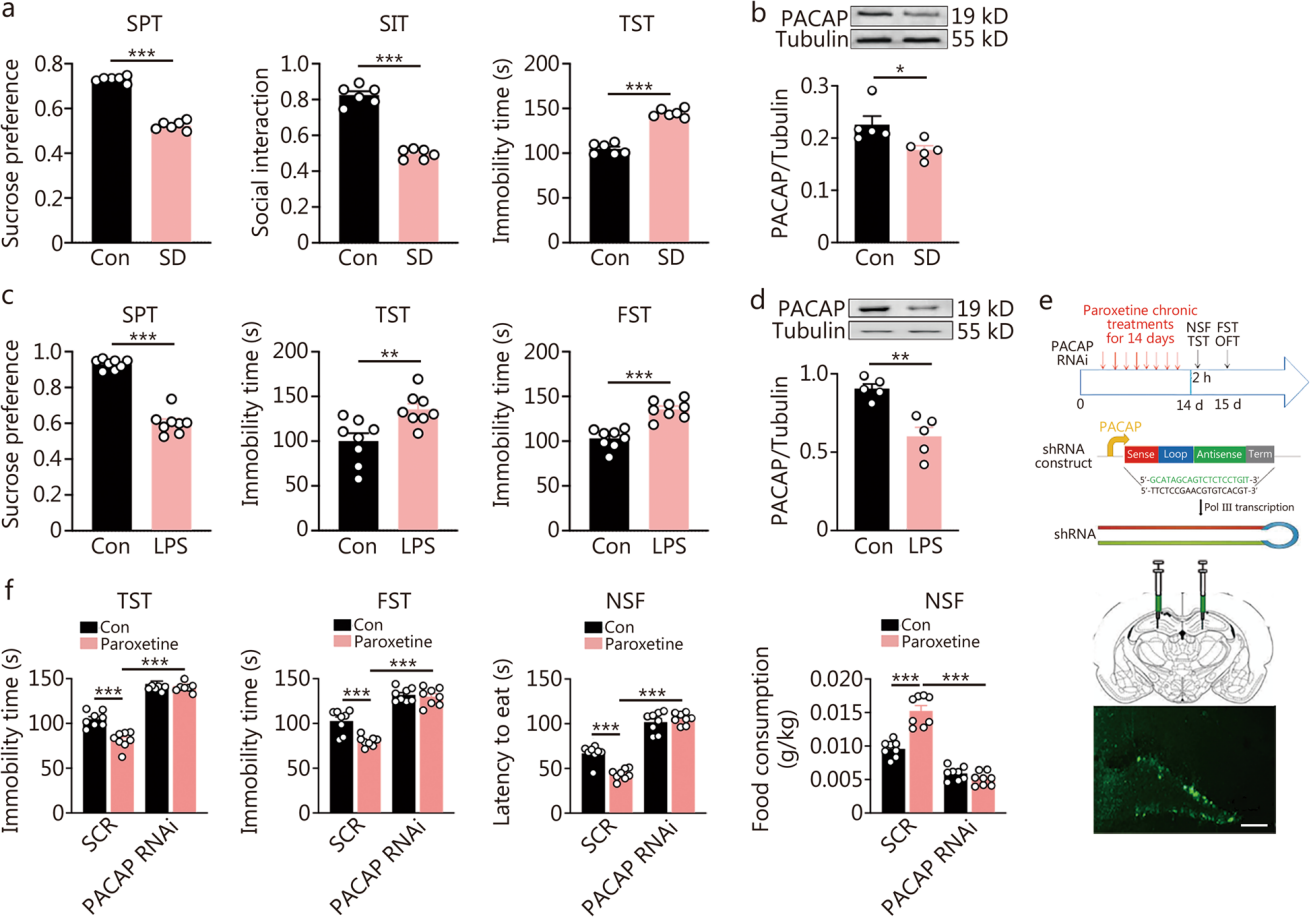
Social defeat (SD) stress or lipopolysaccharide (LPS) injections attenuated the hippocampal pituitary adenylate cyclase-activating polypeptide (PACAP) levels, and knockdown of the dentate gyrus (DG) PACAP induced depression-like behaviors. Following SD exposure (**a**, **b**), LPS injections (**c**, **d**), or control (Con) condition, animals received behavioral tests on the same day in order after the termination of the stress protocol. The brains were harvested to test the expression of PACAP in the hippocampus. **a** Behavioral test following SD for sucrose preference in the sucrose preference test (SPT); the ratio of social interaction times in the social interaction test (SIT); and immobility time in the tail suspension test (TST) (*n* = 6). **b** The expression of PACAP in the hippocampus following SD (*n* = 5). **c** Behavioral tests following LPS for sucrose preference in SPT, immobility time in TST, and immobility time in forced swimming test (FST) (*n* = 8). **d** The expression of PACAP in the hippocampus following LPS injections (*n* = 5). *t*-test, ^*^P < 0.05, ^**^P < 0.01, ^***^P < 0.001. **e** Experimental timeline for tests after bilateral hippocampal dentate gyrus (DG) transfected with virus containing PACAP RNAi or scramble control sequence (SCR) and the design of PACAP RNAi construct and a representative figure for the bilateral hippocampal DG of viral transfection (scale bar = 100 μm). **f** The behavioral comparisons between PACAP knockdown and SCR mice, including immobility time in the TST, immobility time in FST, and latency to eat and food consumption in the novelty-suppressed feeding (NSF) test [*F* (1, 28) = 12.78, *P* = 0.0013; *F* (1, 28) = 9.638, *P* = 0.0043; *F* (1, 28) = 20.25, *P* = 0.0001; *F* (1, 28) = 39.69, *P* < 0.0001] (*n* = 8). Two-way ANOVA, ^***^P < 0.001

To examine the association between lower levels of hippocampal PACAP and depression, we employed two different depression models, a psychosocial stress protocol involving SD (Fig. 2a, b) and a systematic stress protocol using repeated LPS-injection (Fig. 2c). Following exposure to SD, mice showed depression-like responses in the SPT, social interaction test (SIT), and immobility time in the TST (Fig. 2a). SD significantly decreased expression levels of hippocampal PACAP in the hippocampus (Fig. 2b). Similarly, after 4 d of LPS injections, mice exhibited depression-like responses in the SPT, TST and FST (Fig. 2c). LPS significantly reduced expression levels of PACAP in the hippocampus (Fig. 2d).

To further address whether reduced levels of hippocampal PACAP contributed to depressive behaviors and were responsible for the onset of antidepressant response following chronic paroxetine treatment, we investigated the effects of downregulating PACAP^DG^ through intra-DG knockdown of *PACAP* expression (Fig. 2e, f; the verification of the knockdown efficacy shown in Additional file 1: Fig. S1a). Behavioral tests were carried out in mice with bilateral DG viral infection targeting RNAi against PACAP or scrambled control after receiving paroxetine treatments for 14 d (Fig. 2e). In the NSF test, the latency to feed increased while the amount of consumed food decreased (Fig. 2f). The knockdown also increased immobility time in TST and FST (Fig. 2f). Furthermore, the knockdown blunted all of the antidepressant responses measured by TST, FST, and NSF post-chronic paroxetine (Fig. 2f). Time spent in the center and total distance traveled during OFT did not change due to either knockdown or paroxetine administration (Additional file 1: Fig. S1b).

### Optogenetic activation or inhibition of DG PACAP-expressing neurons bidirectionally regulated depression-related behaviors

**Fig. 3:**
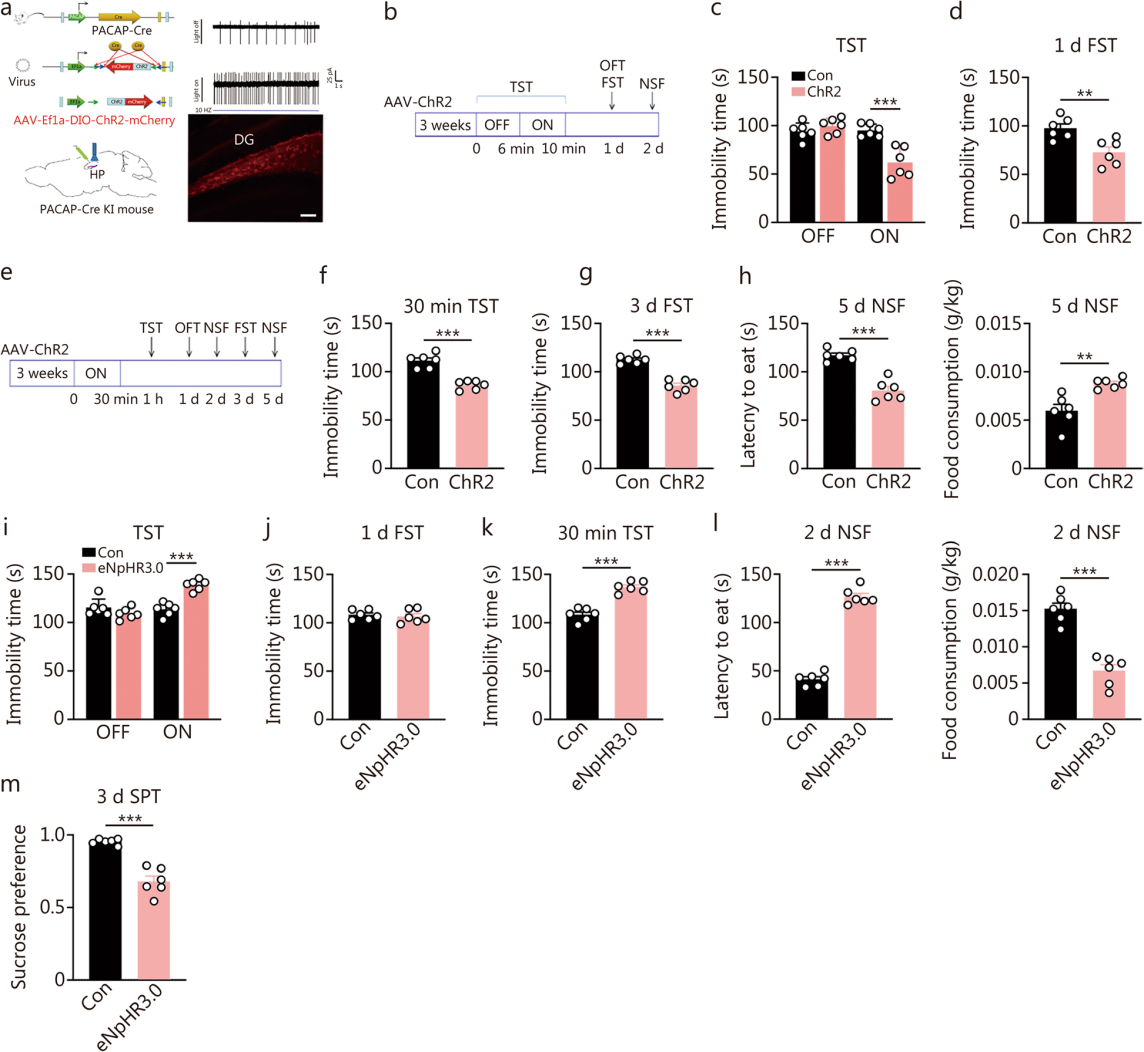
Optogenetic stimulation or inhibition of dentate gyrus (DG) pituitary adenylate cyclase-activating polypeptide (PACAP)-expressing neurons bidirectionally regulated depression-related behaviors immediately and lastingly. **a** The scheme of optogenetic manipulation of DG PACAP-expressing neuronal subtypes, the histological verification of the AAV transfection sites (scale bar = 50 μm), and electrophysiological verification of the neuronal activity. **b** The experiment was designed for a transient (4 min) activation of PACAP neurons. **c** Depression-related behavioral effects by 4 min opto-stimulation in the ChR2 and scramble control (Con) mice, including immobility time in the tail suspension test (TST) before (light off) and during (light on) 4 min opto-stimulation [*F* (1, 20) = 21.45, *P* = 0.0002]. Two-way ANOVA, ^***^P < 0.001. **d** Immobility time in the forced swimming test (FST) tested at 1 d after the opto-stimulation (*n* = 6). **e** The experiment was designed for a long-time (30 min) optogenetic activation of PACAP-expressing neurons. **f** Immobility time in the TST at 30 min after the opto-stimulation. **g** Immobility time in FST at 3 d after the opto-stimulation. **h** Latency to feed and food consumption in the novelty-suppressed feeding (NSF) test at 5 d post-opto-stimulation (*n* = 6). *t*-test, ^**^P < 0.01, ^***^P < 0.001. **i** Immobility time in TST was measured before (light off) and during 4 min opto-inhibition (light on) [*F* (1, 20) = 31.73,* P* < 0.0001]. Two-way ANOVA, ^***^P < 0.001. **j** Immobility time in FST measured at 1 d post 4 min opto-inhibition. **k** Immobility time in TST following 30 min opto-inhibition. **l** Latency to feed and food consumption in NSF test. **m** sucrose preference test (SPT) at 3 d post-opto-inhibition (*n* = 6). *t*-test, ^***^P < 0.001

The above findings demonstrate that hippocampal levels of PACAP play a crucial role in determining depression/antidepressant behaviors. PACAP-expressing neurons in the hippocampus are predominantly located in the DG, suggesting their endogenous and bidirectional regulation of depression-like behaviors. To investigate this possibility, we conducted optogenetic experiments to either stimulate or inhibit PACAP^DG^ neurons in PACAP-directed Cre recombinase (Adcyap1-2A-Cre) knock-in mice. Bilateral delivery of AAV-mediated ChR2 or control vectors was performed stereotaxically into the DG (Fig. 3a-h; Additional file 1: Fig. S2a-e), with histological verification of injection sites as indicated in Fig 3a. Subsequently, light stimulation was administered for varying durations. In the short-light protocol (Fig. 3b-d), animals were subjected to a TST for 10 min, with light stimulation provided during the final 4 min (Fig. 3c). Notably, ChR2 mice exhibited significantly reduced TST immobility time compared to scramble control mice during the 4 min light-on period but showed no difference during the preceding 4 min light-off period (Fig. 3c; min-by-min analysis presented in Additional file 1: Fig. S2a). There was still an antidepressant effect in FST 1 d after opto-activation (Fig. 3d), but the antidepressant effect did not persist beyond that point as observed in the NSF test on the following day (Additional file 1: Fig. S2d). In contrast, under the long-light protocol, behavioral tests were conducted following a 30 min opto-activation period (Fig. 3e-h). Antidepressant effects were evident at different time points post-opto-activation including TST at 30 min, NSF at 2 d, FST at 3 d, and NSF at 5 d respectively (Fig. 3e-h; Additional file 1: Fig. S2e). However, opto-stimulation did not alter OFT behavior (Additional file 1: Fig. S2b, c).

Conversely, the optogenetic inhibition effects were assessed using AAV-mediated eNpHR3.0 or control vectors in the PACAP knock-in mice (Fig. 3i-m; Additional file 1: Fig. S3a-c). The inhibitory effects of optogenetics were essentially opposite to its activation counterpart: a 4-minute light stimulation resulted in an increase in immobility time during TST (Fig. 3i; Additional file 1: Fig. S3a). One day following this 4-minute opto-inhibition, no significant difference was found between the two groups in FST immobility time (Fig. 3j). When subjected to a prolonged period of optogenetic inhibition for 30 min, mice showed depression-like behavior during TST at 30 min, NSF at 2 d, and SPT at 3 d post-inhibition (Fig. 3k-m). However, there was no alteration observed in terms of total distance or time spent in the central area during OFT (Additional file 1: Fig. S2b, c).

### PACAP quickly resulted in CaMKII/eEF2 inhibition and mTOR activation

**Fig. 4:**
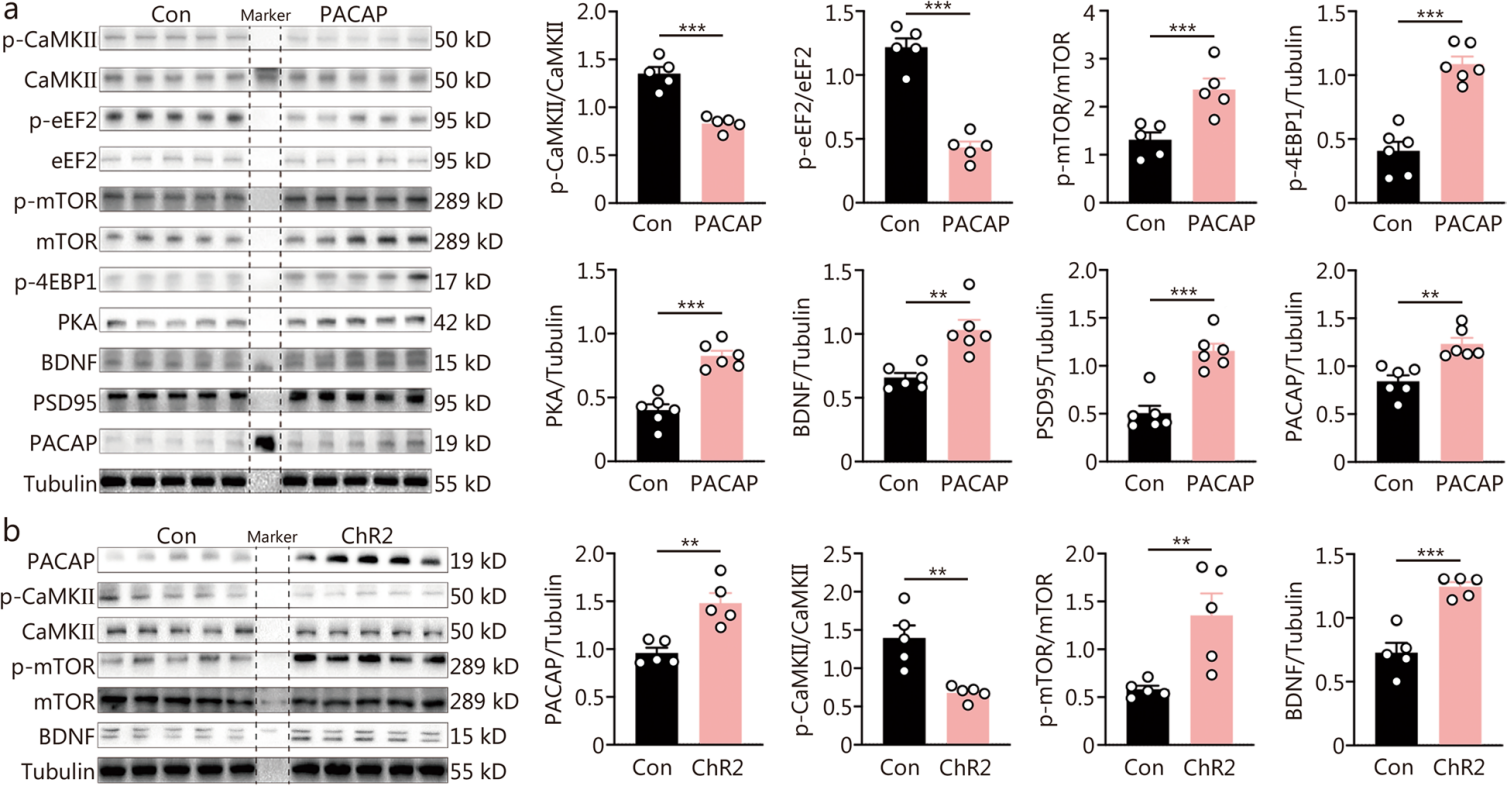
Intra-dentate gyrus (DG) pituitary adenylate cyclase-activating polypeptide (PACAP) infusion regulated neuroplasticity signaling of CaMKII/mTOR/eEF2. **a** Expressions of p-CaMKII/CaMKII, p-eEF2/eEF2, p-mTOR/mTOR, p-4EBP1, PKA, BDNF, PSD95, and PACAP at 30 min following intra-DG PACAP or control (Con) infusion (*n* = 5). **b** Cell signaling at 30 min post a 4-minute optogenetic activation of PACAP-ergic neurons in the DG between the ChR2 group and scramble control (Con) group, including expressions of PACAP, p-CaMKII/CaMKII, p-mTOR/mTOR, BDNF (*n* = 5). *t*-test, ^**^P < 0.01, ^***^P < 0.001. CaMKII calcium/calmodulin-dependent protein kinase II, mTOR mammalian target of rapamycin, eEF2 eukaryotic elongation factor 2, 4EBP1 4E-binding protein 1, PKA protein kinase A, BDNF brain-derived neurotrophic factor, PSD95 postsynaptic density protein 95

The instant enhancement of hippocampal neuroplasticity underlying rapid antidepressant response critically relies on the signaling pathways of eEF2, mTOR, and CaMKII. Therefore, we investigated whether PACAP modulated these signaling pathways. Following DG PACAP infusion for 30 min, the phosphorylation of CaMKIIa at threonine 286 (T286) in the hippocampus was significantly downregulated (Fig. 4a). Similarly, the phosphorylation of eEF2 at threonine 56 (T56) was also significantly decreased. In contrast, the phosphorylation of mTOR at serine 2448 (S2448), as well as its downstream target protein 4E-binding protein 1 (4EBP1) at threonines 37/46 were upregulated (Fig. 4a). Additionally, the expressions of BDNF and synaptic protein PSD95 were increased (Fig. 4a). Furthermore, PACAP and its classic downstream signaling pathway PKA were upregulated as well (Fig. 4a). Similarly, 4-minute optogenetic stimulation of DG PACAP-expressing neurons resulted in increased expression levels of PACAP, p-mTOR and BDNF while decreasing CaMKII signaling after a post-stimulation period of 30 min (Fig. 4b). Interestingly, 1 h after DG PACAP infusion, there was an upregulation in the phosphorylated expression level at T286 for CaMKIIa signaling; however, the inhibition on T56 phosphorylation level for eEF2 remained unchanged (Additional file 1: Fig. S4).

### Pre-activation of CaMKII signaling selectively attenuated PACAP-induced rapid antidepressant response and mTOR/eEF2 signaling

**Fig. 5:**
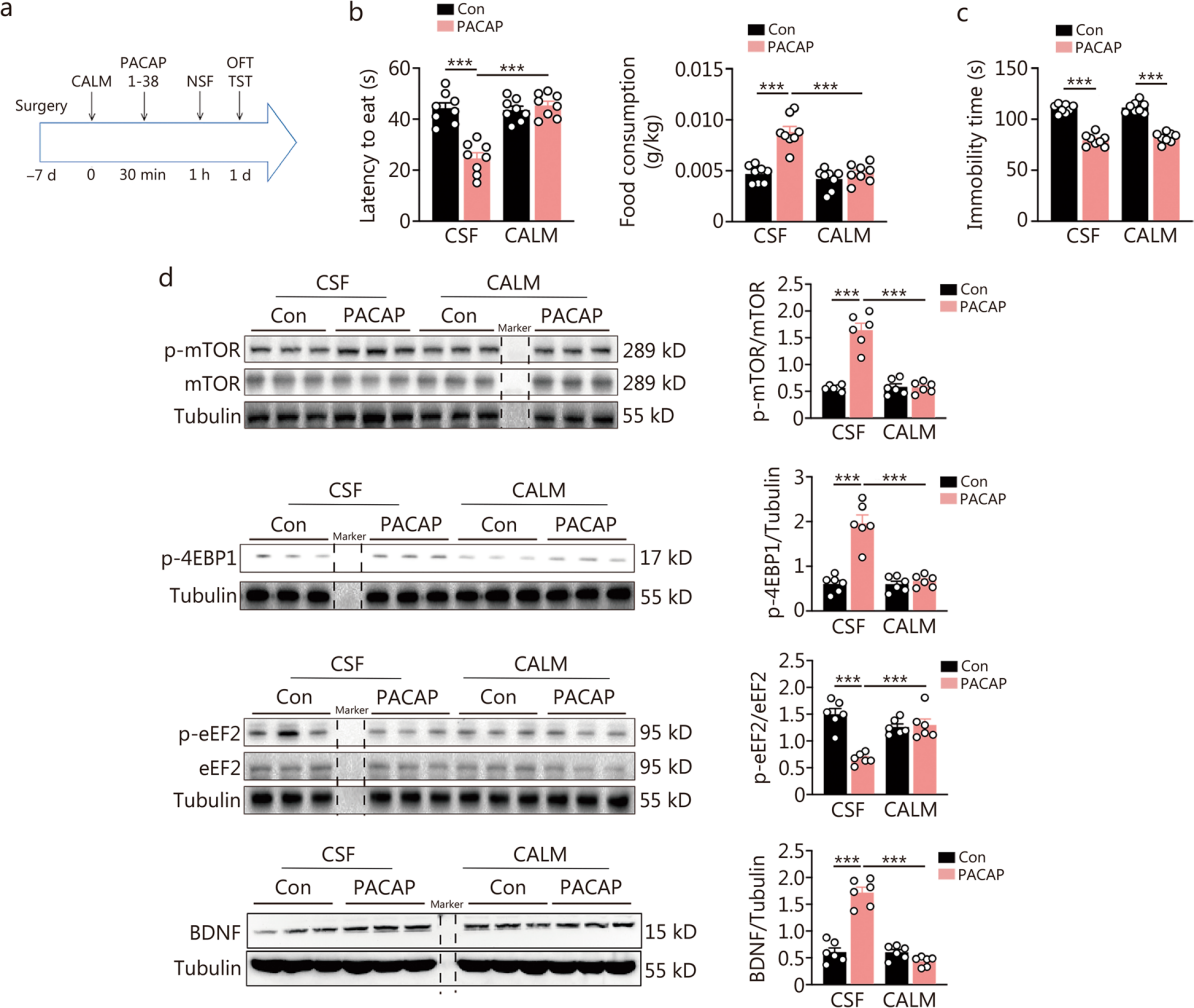
Effects of intra-dentate gyrus (DG) blockade of CaMKII signaling inhibition on the rapid onset of antidepressant response and protein synthesis-related signaling induced by pituitary adenylate cyclase-activating polypeptide (PACAP). **a** Experimental procedure for intra-DG microinfusion of CaMKII agonist (CALM) or cerebrospinal fluid (CSF) followed by PACAP or control (Con) microinfusion. **b** Latency to feed and food consumption in the novelty-suppressed feeding (NSF) test at 30 min [*F* (1, 28) = 31.91, *P* < 0.0001; *F* (1, 28) = 20.43, *P* = 0.001]. **c** Immobility time in tail suspension test (TST) at day 1 [*F* (1, 28) = 0.144, *P* = 0.707] (*n* = 8). **d** The effects of pre-activation of CaMKII on intra-DG PACAP-induced cell signaling response (*n* = 6). Expressions of p-mTOR/mTOR, p-4EBP1, p-eEF2/eEF2, and BDNF at 30 min after intra-DG PACAP infusion [p-mTOR/mTOR: *F* (1, 20) = 53.35, *P* < 0.0001; p-4EBP1: *F* (1, 20) = 33.65,* P* < 0.0001; p-eEF2/eEF2: *F* (1, 20) = 26.09, *P* < 0.0001; BDNF: *F* (1, 20) = 79.54, *P* < 0.0001]. Two-way ANOVA, ^***^P < 0.001. CaMKII calcium/calmodulin-dependent protein kinase II, mTOR mammalian target of rapamycin, eEF2 eukaryotic elongation factor 2, 4EBP1 4E-binding protein 1, BDNF brain-derived neurotrophic factor

The initial inhibition of CaMKII signaling, as suggested by a previous study, has been associated with a rapid antidepressant effect [13]. In this study, we observed that PACAP initially inhibits CaMKII signaling. Based on this finding, we hypothesized that the downregulated phosphorylated expression of CaMKII mediates the rapid antidepressant effect of PACAP. To test this hypothesis, we conducted a single bilateral intra-DG infusion of the CaMKII agonist CALM (40 ng/site), followed by an intra-DG infusion of PACAP (0.4 ng/site, Fig. 5a). Pretreatment of CALM prevented the antidepressant effect in the NSF at 30 min post-PACAP administration (Fig. 5b). However, it no longer had an effect on the sustained antidepressant effect of PACAP tested in the TST 1 d later (Fig. 5c). There were no alterations in total distance or time spent in the central area during OFT (Additional file 1: Fig. S5a). Additionally, we investigated the role of CaMKII signaling on other neuroplasticity pathways induced by PACAP. The upregulation of mTOR, 4EBP1 signaling, and BDNF expression, as well as inhibition of eEF2 signaling at 30 min post-PACAP administration, were all blunted by pretreatment with CALM (Fig. 5d). We also examined its effects on expressions of another mTOR downstream signaling molecule P70S6K and synaptic protein PSD95. However, CALM pretreatment did not influence PACAP-induced upregulation of P70S6K and PSD95 expressions (Additional file 1: Fig. S5b).

We confirmed the initial suppression of CaMKII signaling was solely responsible for the rapid, but not persistent, antidepressant effect of PACAP. It is well established that PACAP stimulates the classic PKA-CREB signaling, which may enhance the neuroplasticity at the transcript level, and thus regulate the enduring antidepressant effects of PACAP. To investigate the possibility further, we also examined the effect of pretreatment with a PKA signaling inhibitor H89 (1 µg/site) on both the rapid and persistent antidepressant effects of PACAP (Additional file 1: Fig. S6a-d). This pretreatment did not affect the rapid antidepressant response to PACAP at 30 min in the NSF test (Additional file 1: Fig. S6b). However, it attenuated the antidepressant effects in TST 1 d after PACAP infusion (Additional file 1: Fig. S6c). There were no changes observed in total distance or time spent in the central zone during OFT (Additional file 1: Fig. S6d).

### The rapid antidepressant response of ketamine was dependent on PACAP^DG^ signaling activation

**Fig. 6:**
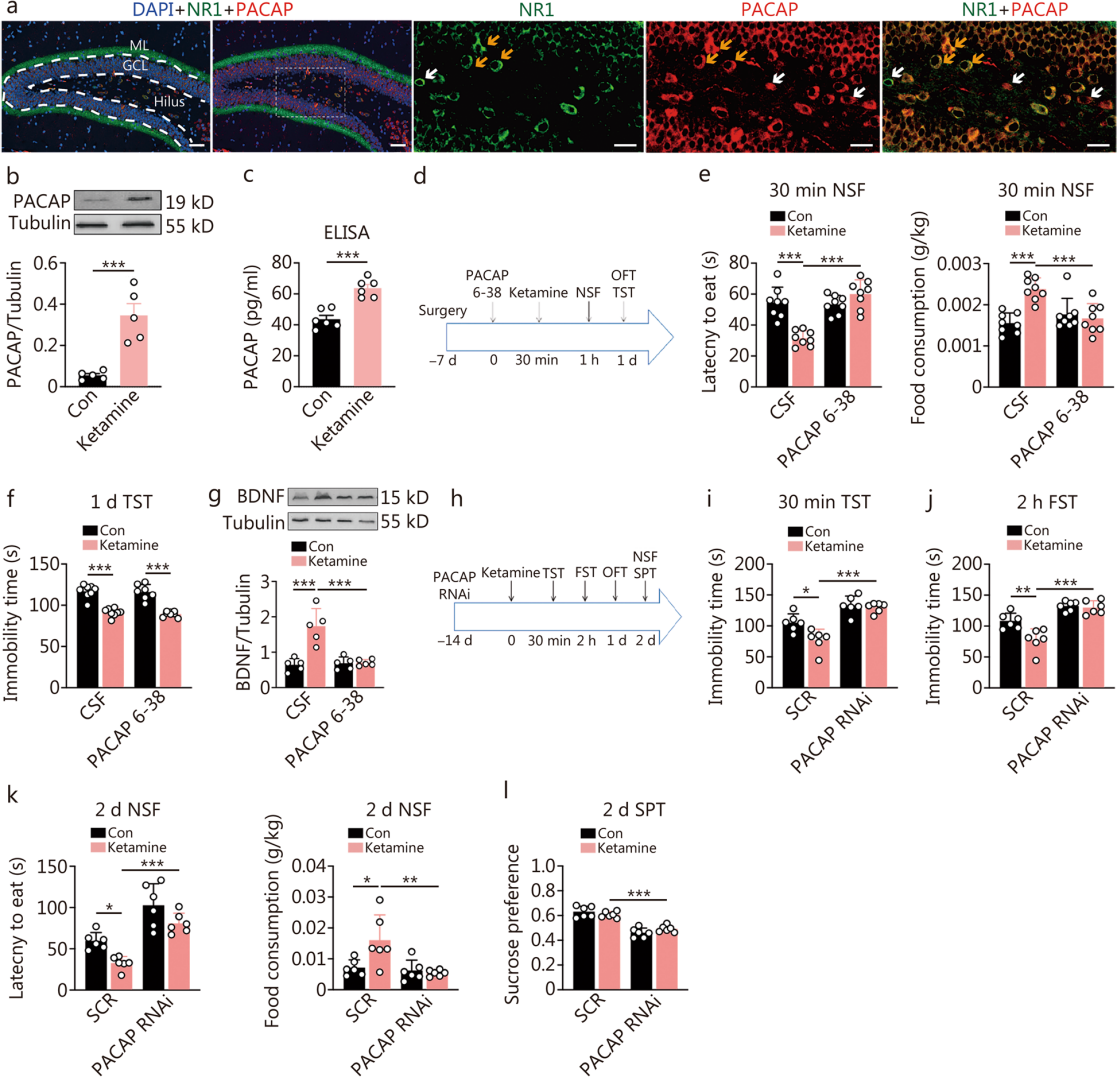
The influence of dentate gyrus (DG) pituitary adenylate cyclase-activating polypeptide (PACAP) signaling on the rapid onset of the antidepressant response of ketamine. **a** Co-immunostaining of PACAP (red color) and NMDAR subunit NR1 (green color) in the DG. The cell nuclei were stained with DAPI (blue color). Yellow arrows point to representative cells co-labeled with PACAP and NR1, and white arrows point to those singly labeled (scale bars = 50 μm and 20 μm, respectively). **b** The peptide expression of PACAP in the hippocampus at 30 min post a single administration of ketamine or control (Con) by Western blotting analysis (*n* = 5). **c** The peptide expression of PACAP in the hippocampus at 30 min post a single administration of ketamine or Con by ELISA analysis (*n* = 6). *t*-test, ^***^P < 0.001. **d** The experiment design for intra-DG microinfusion of PACAP 6-38 or cerebrospinal fluid (CSF). **e** Latency to eat and food consumption in novelty-suppressed feeding (NSF) at 30 min post ketamine [*F* (1, 28) = 28.93, *P* < 0.0001; *F* (1, 28) = 16.20, *P* = 0.0004]. Two-way ANOVA, ^***^P < 0.001. **f** Immobility time in the forced swimming test (FST) at 1 d post ketamine [*F* (1, 28) = 0.038, *P* = 0.847] (*n* = 8). **g** The expression of hippocampal BDNF at 30 min post-intra-DG PACAP 6-38 microinfusion and ketamine [*F* (1, 16) = 18.23, *P* < 0.001] (*n* = 5). **h** The experiment was designed for transfected with a virus containing PACAP RNAi or scramble control sequence (SCR). **i** Immobility time in the tail suspension test (TST) at 30 min post ketamine [*F* (1, 20) = 4.475, *P* = 0.047]. **j** Immobility time in the FST at 2 h post ketamine [*F* (1, 20) = 7.197, *P* = 0.0143]. **k** Latency to eat and food consumption in the NSF at 2 d post ketamine [*F* (1, 20) = 49.86, *P* < 0.0001; *F* (1, 20) = 6.557, *P* = 0.0186]. **l** The percentage of the drinking of sucrose water in the sucrose preference test (SPT) at 2 d post ketamine [*F* (1, 20) = 3.828, *P* = 0.045] (*n* = 6). Two-way ANOVA, ^*^P < 0.05, ^**^P < 0.01, ^***^P < 0.001

After observing similar neuroplasticity signaling changes in CaMKII/mTOR/eEF2/BDNF induced by both PACAP and ketamine, we investigated whether the rapid antidepressant response of ketamine was mediated by PACAP in DG. Initially, immunofluorescent studies were conducted to determine if PACAP-expressing neurons in DG also expressed the N-methyl-D-aspartic acid receptor (NMDAR). Therefore, co-immunostaining of PACAP with the NMDAR subunit 1 (NR1) was performed. Our findings revealed that the PACAP-immunopositive neurons were primarily located in the DG hilus, and the majority of them were co-immunostained with NR1. Notably, there was a strong co-labelling of PACAP and NR1 in the terminals surrounding the granule cells (Fig. 6a). Subsequently, we examined whether ketamine can quickly upregulate PACAP expression in the hippocampus. We found that at 30 min post-administration of an optimal dose for rapid antidepressant response (30 mg/kg) [17], there was an increase in PACAP peptide expression (Fig. 6b, c), but not gene expression in the hippocampus (Additional file 1: Fig. S7a). To assess the role of PACAP^DG^, bilateral intra-DG infusion with the antagonist PACAP 6-38 (40 pg/site) was performed 30 min before a single dose of ketamine administration (Fig. 6d-f). The results showed that this antagonist attenuated the antidepressant response induced by ketamine at 30 min post-administration as measured by NSF test performance (Fig. 6e). However, blockade with this antagonist did not affect the sustained antidepressant effect tested using FST at 1 d post-ketamine administration (Fig. 6f). Previous studies have demonstrated that ketamine quickly triggered the synthesis of proteins/peptides including BDNF [10, 17]. Based on our hypothesis that it is mediated through the PACAP signaling pathway, we further investigated how intra-DG antagonism of PACAP influences ketamine-induced BDNF expressions. As expected, the upregulation of BDNF expression by ketamine was attenuated (Fig. 6g). We also tested the dependence of the action of ketamine on CaMKII signaling inhibition (Additional file 1: Fig. S7b), and found that pretreatment with CALM in intra-DG to activate CaMKII signaling prevented the rapid antidepressant response in the NSF test at 30 min post-ketamine (Additional file 1: Fig. S7c). The pretreatment no longer had an effect on the sustained antidepressant effect in the TST at 2 h post-ketamine (Additional file 1: Fig. S7d). At 30 min post-ketamine, the blockade of CaMKII inhibition also attenuated the upregulation of BDNF by ketamine (Additional file 1: Fig. S7e).

The impact of PACAP^DG^ downregulation on ketamine-induced antidepressant behaviors was further evaluated by employing PACAP expression knockdown (Fig. 6h-l). Behavioral tests were carried out in mice with bilateral DG viral infection of RNAi targeting PACAP or scrambled control after a single dose of ketamine (Fig. 6h). The knockdown completely abolished all of the antidepressant responses tested at different time points following ketamine administration, including TST at 30 min, FST at 2 h, NSF at 2 d and SPT at 2 d post-ketamine (Fig. 6i-l). Intra-DG PACAP antagonism or PACAP knockdown treatment did not alter OFT behavior along with ketamine administration (Additional file 1: Fig. S8a, b).

## Discussion

The present study aimed to investigate the role of hippocampal DG PACAP signaling in regulating the rapid onset of antidepressant response. We observed a concurrent increase in hippocampal PACAP expression with the initiation of antidepressant effect following chronic paroxetine administration. Furthermore, enhancing PACAP signaling through intra-DG infusion or optogenetic activation of PACAP^DG^ induced a rapid and persistent antidepressant response. Conversely, we found reduced levels of hippocampal PACAP in mice exhibiting depression-like symptoms induced by LPS or SD stress. Additionally, forced attenuation of PACAP signaling through intra-DG *PACAP* knockdown or optogenetic inhibition of PACAP-expressing neurons resulted in robust depression-like behaviors. Our findings suggest that the inhibitory effect of PACAP on CaMKII is crucial for mTOR/eEF2/BDNF neuroplasticity signaling, which plays a critical role in mediating the rapid antidepressant response. Finally, we confirmed that the activation of PACAP^DG^ is necessary for both the rapid enhancement of BDNF and the rapid antidepressant response elicited by ketamine.

Our findings suggest that the DG PACAP may be a novel target for rapid antidepressant response. Studies indicate that these PACAP-ergic neurons are mossy cells, which primarily project axons to granule cells where the PACAP receptor 1 (PAC1) is highly expressed [28, 29]. While adult-born granule cells in the DG have been extensively studied for their role in depression regulation, the involvement of regulatory mossy cells in depression has been relatively understudied. We discovered that even a brief inhibition of PACAP neuronal activity in the DG induced immediate depression-like behavior, whereas longer optogenetic inhibition or knockdown of PACAP^DG^ levels resulted in long-term depression-like behavior. These findings were consistent with the results of previous studies on CD-1 background mice lacking PACAP [14, 20, 21]. Similarly, reduced hippocampal levels of PACAP were observed in two distinct stress models associated with depression: SD psychosocial stress model and systematic stress model using LPS injection. The latter elicits depression-like behavior plausibly through preferential conversion of tryptophan to kynurenine signaling [30, 31], a mechanism shared for depression and pain. Therefore, the LPS injection model may potentially augment the susceptibility to pain [30]. Together, deficiency of PACAP^DG^ likely contributes to the pathophysiology of depression (Figs. 2 and 3i-m). Conversely, enhancement of PACAP^DG^ elicited a rapid antidepressant response as indicated by instant improvement in performance on the NSF test, which could be achieved through acute treatment with ketamine or chronic administration of SSRIs [32, 33] (Figs. 1a and 6e). We observed an immediate induction of PACAP expression by ketamine (Fig. 6b, c), and the onset of NSF improvement coincided with the upregulation of PACAP following chronic treatment with paroxetine (Fig. 1b, c). Furthermore, the rapid enhancement in NSF performance, but not behavior despair, induced by certain natural compounds was dependent on hippocampal PACAP^DG^ activation [13]. It has been demonstrated that the cornu ammonis area 3 (CA3) to cornu ammonis area 1 (CA1) projection in the hippocampus is required for rapid onset of antidepressant response elicited by ketamine [34]. Given that DG serves as the initial signal processing station within the hippocampus, our current findings suggest that PACAP^DG^ may act prior to the CA3 – CA1 circuit to initiate signal processing in facilitating a rapid antidepressant response to ketamine [35].

We first revealed that initial inhibition of CaMKII signaling was responsible for the onset, but not the subsequent maintenance of antidepressant activity. A previous study showed a CaMKII signaling antagonist induced antidepressant activity [12], which is consistent with our finding that PACAP initially inhibited CaMKII signaling and pretreatment with a CaMKII agonist attenuated the rapid antidepressant activity of PACAP (Fig. 5b). Previous research has demonstrated that initial inhibition of eEF2 enhances the expression of proteins/peptides including BDNF, which is necessary for the rapid antidepressant response to ketamine [9]. Our results suggest that the eEF2 inhibition occurs after CaMKII inhibition (Fig. 5d). Furthermore, we provided evidence indicating that CaMKII inhibition also led to mTOR activation (Fig. 5d), through which protein synthesis was promoted by CaMKII inhibition in an eEF2-independent or -dependent manner [36]. Overall, both transient and initial inhibitions of CaMKII signaling regulated eEF2 and mTOR signaling pathways and played a central role in promoting protein synthesis and neuroplasticity underscoring rapid antidepressant response. Some studies have observed downregulation of calmodulin activity, an activator of CaMKII signaling, following the activation of PACAP. It is possible that PACAP signaling activation inhibits CaMKII via suppression of the Ca^2+^ influx [37]. Further study should address how PACAP neurotransmission suppresses Ca^2+^ influx/CaMKII signaling and then influences mTOR/eEF2 signaling pathways. ERK1/2 signaling plays an important role in rapid-acting antidepressant effects as it acts upstream to mTOR signaling, and can be activated by PACAP [8, 38]. The involvement of ERK is currently being investigated.

The rapid upregulation of hippocampal BDNF is critical for enhancing neuroplasticity, which is responsible for the rapid antidepressant response to ketamine [9]. The expression of BDNF peptide after chronic SSRI treatment was also associated with the antidepressant response [39]. In this study, we demonstrated that ketamine upregulated BDNF in a PACAP signaling-dependent manner (Figs. 4, 6g), suggesting that ketamine first enhances PACAP peptide expressions, likely in the PACAP-expressing neurons (Figs. 4b and 6b, c), and subsequently activates PACAP neurotransmission in granule cells in which the PACAP receptors PAC1 are highly expressed [40]. The activation of PACAP signaling further promoted BDNF expression through CaMKII/eEF2/mTOR signaling (Fig. 5d). It should be noted that an immediate upregulation of BDNF is required for the rapid antidepressant response to ketamine, but BDNF levels can continue to increase over time. We demonstrated here that blockade of PKA signaling attenuates the persistent, but not the rapid, antidepressant response to PACAP (Additional file 1: Fig. S6a-d). Additionally, the early maintenance of antidepressant response to ketamine depends on PKA-CREB-BDNF signaling (unpublished observation). It is conceivable that PACAP may also contribute to persistent increased BDNF expression through classic PKA-CREB signaling regulation of BDNF transcription. Similarly, the synaptic protein PSD95 expression can be rapidly upregulated via eEF2/mTOR signaling and persistently via PKA-CREB signaling by PACAP. Our findings suggest that hippocampal PACAP signaling may function as a master regulator mediating both rapid and persistent upregulation of BDNF and synaptic protein expressions, respectively, thereby contributing to their respective rapid and persistent antidepressant effects.

**Fig. 7:**
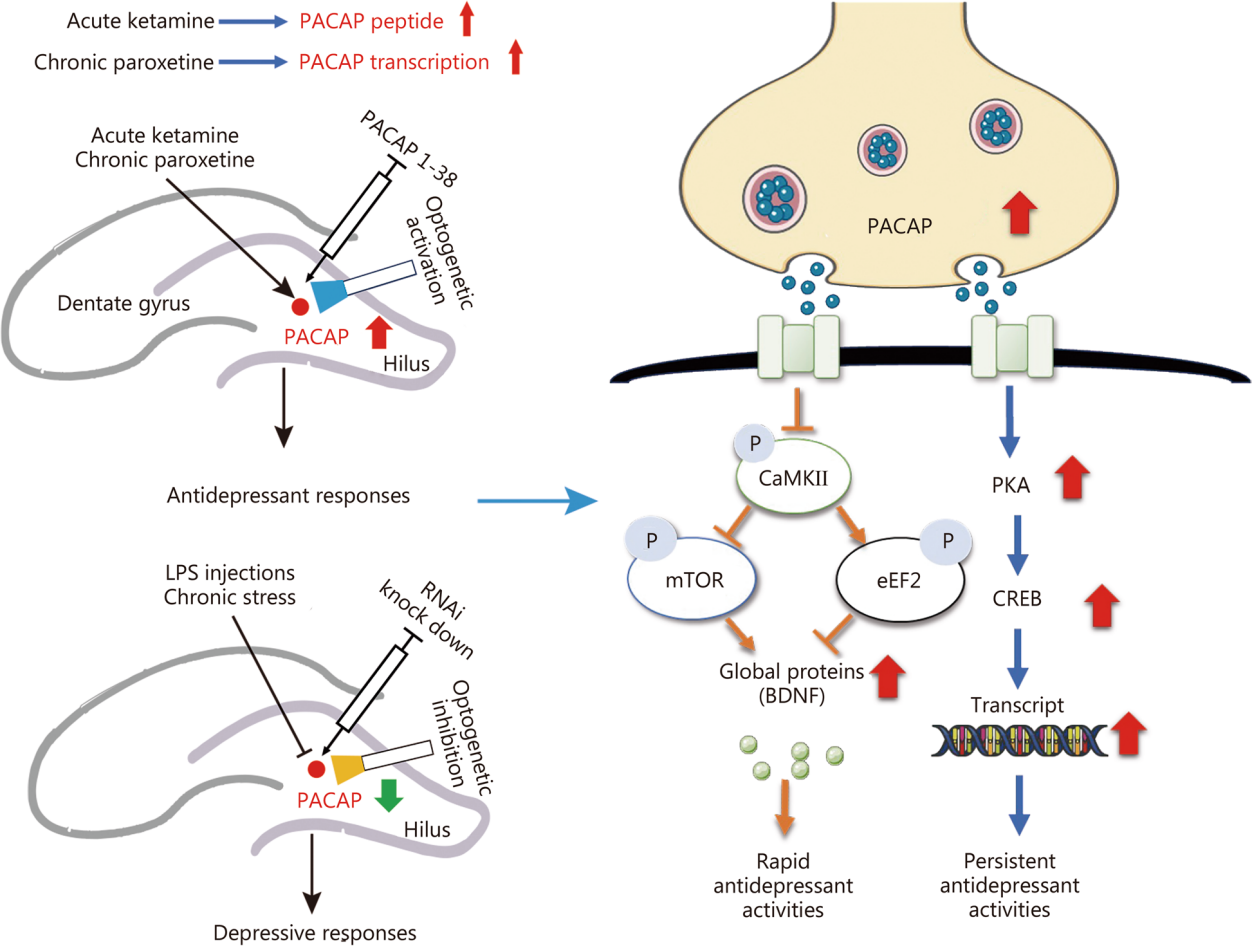
The schematic diagram for hippocampal dentate gyrus (DG) pituitary adenylate cyclase-activating polypeptide (PACAP) regulation of the rapid and persistent antidepressant response. Activating hippocampal DG PACAP signaling produces the onset of antidepressant response, following a repeated treatment of the delayed-onset antidepressant paroxetine to increase the PACAP transcription, or acute treatment of ketamine to instantly enhance PACAP peptide expression. PACAP signaling activation initially inhibits CaMKII signaling, which controls the subsequent eEF2-mTOR-BDNF signaling for eliciting the rapid antidepressant response. PACAP activation of PKA-CREB signaling was involved in the following persistent antidepressant response. CaMKII calcium/calmodulin-dependent protein kinase II, mTOR mammalian target of rapamycin, eEF2 eukaryotic elongation factor 2, PKA protein kinase A, BDNF brain-derived neurotrophic factor, LPS lipopolysaccharide, CREB cAMP-response element binding protein

The present study demonstrates that PACAP-expressing neurons in the DG of the hippocampus bi-directionality regulate depression-like behaviors. The rapid and persistent antidepressant activity is mediated by distinct signaling pathways induced by PACAP. A schematic diagram illustrating this concept is presented in Fig. 7. The onset of antidepressant response requires activation of the PACAP signaling, which enhances BDNF expression through inhibition-controlled mTOR/eEF2 signaling mediated by CaMKII, leading to the rapid antidepressant response. Additionally, activation of the classic PKA-CREB signaling pathway by PACAP is necessary for sustaining the antidepressant response over time. In contrast to prolonged treatment with paroxetine required gene expression changes in PACAP, acute administration of ketamine rapidly upregulates post-transcriptional levels of PACAP. This difference may explain why paroxetine has a slow onset while ketamine exhibits a fast onset in producing an antidepressant effect [26]. A previous study has shown increased expression of PACAP in the hippocampal culture treated with some other SSRIs; however, it remains to be determined if this finding can be generalized to all types of antidepressants [23]. Nonetheless, the present study identifies DG as a key regulatory substrate where activated PACAP-expressing neurons trigger the onset of an antidepressant response.

## Conclusions

This study revealed that inhibition of PACAP in the hippocampal DG results in depression-like behaviors. Activation of PACAP signaling in the hippocampus induces the onset of antidepressant response, necessitating repeated treatment with the delayed-onset antidepressant paroxetine, while only acute treatment with the rapid-acting prototype drug ketamine is required. The initial activation of PACAP signaling inhibits CaMKII signaling, which subsequently regulates eEF2-mTOR-BDNF signaling to elicit a rapid antidepressant response (Fig. 7). The study proposes a strategy to expedite the onset of an antidepressant response by rapidly enhancing the attenuated levels of hippocampal PACAP observed in depression.

### Supplementary Information

**Additional file 1:** Materials and methods. **Fig. S1** Quantitative real-time polymerase chain reaction (PCR) analysis and open field test (OFT) after pituitary adenylate cyclase-activating polypeptide (PACAP) RNAi micro-infusion. **Fig. S2** Min-by-min analysis of the immobility time in the tail suspension test (TST) before and during optogenetic stimulation of pituitary adenylate cyclase-activating polypeptide (PACAP)-expressing neurons in the hippocampal dentate gyrus. **Fig. S3** Min-by-min analysis of the immobility time in the tail suspension test (TST) before and during optogenetic inhibition of pituitary adenylate cyclase-activating polypeptide (PACAP)-expressing neurons in the hippocampal dentate gyrus. **Fig. S4** Pituitary adenylate cyclase-activating polypeptide (PACAP) infusion regulated calcium/calmodulin-dependent protein kinase II (CaMKII)/eukaryotic elongation factor 2 (eEF2) signaling. **Fig. S5** The effects of intra-dentate gyrus (DG) microinfusion of calcium/calmodulin-dependent protein kinase II (CaMKII) agonist (CALM) on the immediate or lasting antidepressant activity of pituitary adenylate cyclase-activating polypeptide (PACAP). **Fig. S6** Intra-HDG microinfusion of protein kinase A (PKA) antagonist H89 on the immediate or lasting antidepressant activity of PACAP. **Fig. S7** The association of pituitary adenylate cyclase-activating polypeptide (PACAP) and related signaling with ketamine. **Fig. S8** Intra-dentate gyrus (DG) pituitary adenylate cyclase-activating polypeptide (PACAP) antagonism or PACAP knockdown treatment did not alter open field test (OFT) behavior along with ketamine administration.

## Data Availability

The datasets generated during the current study will be available from the corresponding author upon reasonable request.

## Abbreviations

BDNF: Brain-derived neurotrophic factor
CaMKII: Calcium/calmodulin-dependent protein kinase II
CA3: Cornu ammonis area 3
cAMP: Cyclic adenosine monophosphate
CREB: cAMP-response element binding protein
ChR2: Channelrhodopsins 2
DG: Dentate gyrus
eEF2: Eukaryotic elongation factor 2
FST: Forced swimming test
LPS: Lipopolysaccharide
MDD: Major depressive disorder
mTOR: Mammalian target of rapamycin
NSF: Novelty suppressed feeding test
OFT: Open field test
PACAP: Pituitary adenylate cyclase-activating polypeptide
PKA: Protein kinase A
SPT: Sucrose preference test
SSRIs: Selective serotonin reuptake inhibitors
TST: Tail suspension test
