# Supplementary material for: Hippocampal PACAP signaling activation triggers a rapid antidepressant response
Source: Mil Med Res. 2024 Jul 23;11:49. doi: 10.1186/s40779-024-00548-1 (PMC11265467; doi:10.1186/s40779-024-00548-1)
Supplement: Supplementary file 1 — Additional file 1: Materials and methods. Fig. S1 Quantitative real-time polymerase chain reaction (PCR) analysis and open field test (OFT) after pituitary adenylate cyclase-activating polypeptide (PACAP) RNAi micro-infusion. Fig. S2 Min-by-min analysis of the immobility time in the tail suspension test (TST) before and during optogenetic stimulation of pituitary adenylate cyclase-activating polypeptide (PACAP)-expressing neurons in the hippocampal dentate gyrus. Fig. S3 Min-by-min analysis of the immobility time in the tail suspension test (TST) before and during optogenetic inhibition of pituitary adenylate cyclase-activating polypeptide (PACAP)-expressing neurons in the hippocampal dentate gyrus. Fig. S4 Pituitary adenylate cyclase-activating polypeptide (PACAP) infusion regulated calcium/calmodulin-dependent protein kinase II (CaMKII)/eukaryotic elongation factor 2 (eEF2) signaling. Fig. S5 The effects of intra-dentate gyrus (DG) microinfusion of calcium/calmodulin-dependent protein kinase II (CaMKII) agonist (CALM) on the immediate or lasting antidepressant activity of pituitary adenylate cyclase-activating polypeptide (PACAP). Fig. S6 Intra-HDG microinfusion of protein kinase A (PKA) antagonist H89 on the immediate or lasting antidepressant activity of PACAP. Fig. S7 The association of pituitary adenylate cyclase-activating polypeptide (PACAP) and related signaling with ketamine. Fig. S8 Intra-dentate gyrus (DG) pituitary adenylate cyclase-activating polypeptide (PACAP) antagonism or PACAP knockdown treatment did not alter open field test (OFT) behavior along with ketamine administration. [file 40779_2024_548_MOESM1_ESM.pdf]

Materials and methods

Chronic stress paradigms and behavioral tests

Chronic mild stress (CMS) paradigm. The CMS protocol was designed to maximize the unpredictable nature of the stressors. Mice were singly housed and received 4 weeks of mild stress. One of the following stressors was administered daily over a period of 3 weeks in random order: food and water deprivation for 24 h, cage tilted at 45° for 24 h, cage shaken (horizontal shakes at high speed, 200 rpm) for 40 min, soiled cage bedding (200 ml water in the sawdust bedding) for 20 h, overnight illumination (total illumination time 36 h), and restraint in a 50 ml tube for 6 h. Control mice were group housed without any stressors given.

Social defeat (SD) procedure and social interaction test (SIT)

The SD procedure was adapted from previous studies with minor modifications. Briefly, for 10 consecutive days, a resident mouse was placed for 10 min into the homecage of a resident Kunming mouse previously screened for aggressive behavior. Following this physical interaction period, a perforated plexiglas partition was inserted in the cage to separate the mice. The procedure was repeated for 10 d, using a new ICR resident mouse each day. Control mice were pair-caged with a conspecific mouse. The SIT was assessed on the day following the last SD session. Social interaction scores were calculated as the amount of time spent near a social target compared with time spent near an empty enclosure for 5 min, using Any-maze software.

Novelty suppressed feeding (NSF) test

In the NSF test, mice were food-deprived for 18 h and then placed into an open field arena (described above). A single pellet of the normal mouse chow was placed in the center of the open-field arena. Each animal was placed at a corner of the arena at the beginning and allowed to explore for 10 min. The trial ended at 10 min or when the mouse chewed on the mouse chow. The latency to begin eating, which is defined as the time until the mouse actively chewed the pellet, was recorded. The amount of food consumed in the home cage control was also measured, as a control measure for appetite.

Tail suspension test (TST)

The TST is widely used in preclinical antidepressant testing. TST was performed with a computerized device allowing four animals to be tested at one time. In a chamber that is acoustically and visually isolated, an individual animal was suspended 50 cm above the floor by adhesive tape placed approximately 1 cm from the tip of the tail. The activity of the animals was videotaped. The total time spent immobile during the last 4 min in a 6 min testing time was analyzed using Any-maze software.

Forced swimming test (FST)

The FST is also one of the most often used behavioral tests for measuring depressive-like responses in rodents. Mice were removed from their home cages, and individually placed into a clear glass tank (40 cm high and 20 cm in diameter) filled with 30 cm of water (22 – 23 °C) and allowed to swim for 6 min. At the end of the test, the animals were removed from the water, dried with a paper towel, and returned to their home cages. The mice were considered immobile when floating in the water without struggling and making only those movements necessary to keep their heads above the water. Total immobility times during the last 4 min of the 6 min testing period were analyzed by ANY-maze software.

Sucrose preference test (SPT)

The SPT followed a published procedure with minor modifications. Briefly, mice were individually housed and exposed to a sucrose solution (1% in tap water, Sigma, St. Louis, MO, USA) for 3 d, followed by 18 h of water deprivation and then exposure to two identical bottles, one filled with 1% sucrose solution and the other with water. The volume of sucrose solution or water was measured after 6 h exposure. Sucrose preference was defined as the ratio of the volume of sucrose versus total volume (sucrose + water) consumed during the 2 h test, normalized with body weight for individual animals.

Open field test (OFT)

The OFT was used to assess the locomotor as well as the anxiety-related behavior in an open area. In the test, spontaneous horizontal locomotor activity was measured in a square arena (40 cm × 40 cm × 35 cm). Mice were tested in a well-illuminated (approximately 300 lux) transparent acrylic cage for 5 min. The activity of mice at the edge and in the central region was tracked. Distance (cm) traveled and the time spent in the central zone were analyzed.

Western blotting analysis

The entire hippocampus was lysed in RIPA buffer containing protease inhibitors and phosphatase inhibitors. Protein concentration was determined colorimetrically by BCA assay. Protein lysates were separated by 10% SDS-PAGE, 15% SDS-PAGE, or 8% SDS-PAGE electrophoresis and were transferred onto polyvinylidene difluoride membranes. After blocking with 3% BSA for 1 h, the membranes were incubated with either anti-pituitary adenylate cyclase-activating polypeptide (PACAP; 1:500, bs-0190R, Bioss, China), anti-eukaryotic elongation factor 2 (eEF2; 1:1000, #2332, Cell Signaling, USA), anti-p-eEF2 (1:1000, #2331, Cell Signaling, USA), anti-calcium/calmodulin-dependent protein kinase II (CaMKII; 1:1000, AF6343, Affinity, USA), anti-p-CaMKII (1:500, AF3493, Affinity, USA), anti-protein kinase A (PKA; 1:1000, #4782, Cell Signaling, USA), anti-

postsynaptic density protein-95 (PSD95; 1:1000, #2057, Cell Signaling, USA), anti-p-4EBP1 (1:1000, #2855, Cell Signaling, USA), anti-mammalian target of rapamycin (mTOR; 1:1000, #2972, Cell Signaling, USA), anti-p-mTOR (1:1000, #2971, Cell Signaling, USA), anti-brain derived neurotrophic factor (BDNF; 1:1000, ab108319, Abcam, USA) or anti-tubulin (1:2000, #5666, Cell Signaling, USA) antibodies at room temperature (26 ℃) for 4 h. Membranes were then washed 30 min with TBST, followed by incubation with horseradish peroxidase-conjugated secondary antibodies for 2 h. The membranes were washed for 30 min with TBST. The blots were visualized using the Immobilon Western Chemiluminescent HRP Substrate, normalized to tubulin.

ELISA

The brain-hippocampus sample was centrifuged at 1000 *g* for 10 min at 4 °C and then was collected and stored at −80 °C. The hippocampus was homogenized in a stabilization buffer (0.05 ng HCl with 0.1% ascorbic acid; 1:10, w/v). The homogenate was centrifuged at 14,000 *g* at 4 °C for 15 min, and then the supernatant was passed through a 0.45 μm centrifugal filter (Merck Millipore, Germany). According to the manufacturer's instructions, the ELISA kit (Biosharp, China) was used to measure the expression of PACAP in the hippocampus.

Immunofluorescence

After each mouse was anesthetized using pentobarbital sodium, perfused, and fixed with 4% paraformaldehyde in PBS, the brain was collected and immersed in 30% sucrose until sunk to the bottom. The brain was sectioned with a cryomicrotome (thickness 30 μm). The brain sections were washed three times with 0.01 mol/L PBS, blocked with 5% bovine serum albumin/0.3% Triton X-100/PBS for 1 h at room temperature, and then added with anti-PACAP primary antibody (1:200, sc-166180, Santa Claus, USA) and anti-N-methyl-D-aspartic acid receptor subunit 1 (NR1) primary antibody (1:500, ab174309, Abcam, USA), 4 ℃ overnight. After being rinsed three times with 0.01 mol/L PBS, Alex Fluor 488 conjugated Goat Anti-Rabbit IgG (H + L) (1:200, ab150077, Abcam, USA) and Alex Fluor 594 conjugated Goat Anti-Mouse IgG (H + L) (1:200, ab150116, Abcam, USA) secondary antibodies were added and incubated for 1 h at room temperature. Following extensive rinses, the brain sections were mounted with glycerol-DAPI media (C1005, Beyotime, China). The immunostaining was examined fluorescently under a microscope (DM2500, Leica, Germany).

Quantitative real-time PCR (RT-PCR) analysis

RNA was isolated from the whole hippocampus (ventral and dorsal) using TRIzol reagent (Invitrogen, USA) and was reverse transcribed to cDNA using the SYBR PrimeScript RT-PCR Kit (TaKaRa, Japan). Quantitative RT-PCR was performed with 1.5 μl of cDNA using the SYBR Green Master Mix reagent (TaKaRa, Japan). The primer showed the following: PACAP: forward 5'-

AGGTGCTGGTGTTGGAATGAATG-3’ and reverse 5’-ATGAGGGCAAGGGTAGGAAGG-3’; GAPDH: forward 5’-AAC GACCCCTTCATTGAC-3’ and reverse 5’-TCCACGACATACTCAGCAC-3’. The fold change in PACAP expression (coding exon) was normalized to GAPDH. The qPCR was carried out based on the manufacturer’s manual. Relative expression values were obtained by the $2^{-\Delta\Delta CT}$ method.

Surgery

Mice were anesthetized using pentobarbital sodium [45 mg/kg; 57-33-0; intraperitoneal injection (i.p.)] and placed on a stereotactic apparatus for surgery. During the operation, all the mice were given continuous oxygen by ventilators for spontaneous breathing. The coordinates for the hippocampus dentate gyrus (DG) were: ML ± 0.2 cm; AP -0.21 cm from Bregma; DV -0.24 cm from dura. In general, no antibiotics or analgesic was used. Animals recovered for 1 week or 3 weeks before microinfusion or optogenetic experimentation, respectively. Histological verification was carried out to only include the data from the mice injected into the designated sites for further analysis.

Microinfusion

After anesthesia, mice were implanted with a 26-gauge stainless steel cannula (RWD, China) into bilateral hippocampi (coordinates: ML ± 0.2 cm; AP -0.21 cm from Bregma; DV -0.24 cm from dura) for intra-DG infusion. A 30-gauge internal injector with a 3 mm projection was placed into the guide cannula, connected via PE tubing to a Hamilton microsyringe driven by a pump (RWD, China). PACAP 1-38 (1.5 – 1200 ng/site), and CALM (40 ng/site) injections were administered over 2 min (0.5 µl/min) and injectors were left in situ for an additional minute to prevent backflow.

Intra-DG viral transfection of shRNA for *PACAP* knockdown, eNpHR3.0, or channelrhodopsins 2 (ChR2)

Both shPACAP (5’-GCATAGCAGTGTCTCCTGTTC-3’) and scrambled control (5’-TTCTCCGAA CGTGTCACGT-3’) were designed by Genepharma Company. The shRNA or scramble sequence was assembled into a lentivirus vector. The virus titer of shPACAP and scrambled control were 3 × 108 TU/ml. After anesthetized, placed in a stereotactic apparatus. Lentivirus for PACAP shRNA with a Hamilton syringe (3 µl/side; 0.5 µl/min) was injected into the hippocampus with coordinates as above mentioned. At least 2 weeks of recovery were required before behavioral tests.

The virus of AAV-DIO-EF1α-eNpHR3.0 or ChR2-mCherry was transfected into a hippocampus. The virus titer of eNpHR3.0 or ChR2 and scrambled control were 3 × 10^8 TU/ml. Virus for eNpHR3.0 with a Hamilton syringe (0.2 µl/side; 0.01 µl /min) were injected into the hippocampus: At least 21 d of recovery were required before behavioral tests. Histological verification was carried

out to only include the data from the mice injected into the designated sites for further analysis.

Hippocampus slice preparation and electrophysiology recording

Brains were quickly removed after anesthesia using isoflurane and horizontal 350 μm hippocampal slices were cut (VT1200S; Leica, Concord, Ontario, Canada) in ice-cold artificial cerebrospinal fluid containing (in mmol/L): sucrose 194; NaCl 29.95; KCl 4.56; $NaHCO_3$ 26; $MgCl_2$ 0.1; NaH_2PO_4 1.17; $CaCl_2$ 1; and glucose 9.99. Slices were collected into an incubation chamber containing artificial cerebrospinal fluid (in mmol/L): NaCl 119; KCl 2.5; $NaHCO_3$ 26; $MgCl_2$ 1.3; NaH_2PO_4 1.0; $CaCl_2$ 2.5; and glucose 11. Both solutions were oxygenated with 95% O_2:5% CO_2. The incubation chamber was placed at room temperature after holding at 32 ℃ for 30 min. Slices remained at room temperature for at least an hour before recording. To confirm eNpHR3.0 expression, whole-cell recordings were obtained from the DG neurons, and action potentials were triggered by blue-light pulses (5 ms, 578 nm, 1 – 4 mW) using a Dual OptoLED (Cairn Research, Faversham, Kent, United Kingdom) delivered through a 40× objective lens. All data acquisition and analysis were done using pCLAMP 10.6 (Axon Instruments, Foster City, California, USA) and the MiniAnalysis program (Synaptosoft, Fort Lee, New Jersey, USA). In all electrophysiological experiments, *n* represents the number of neurons and normally one or two neurons in a slice per animal were used.

Statistical analyses

Mice were randomly divided into designated experimental groups and random numbers were generated using the Microsoft Excel RAND (RRID: SCR_016137) function. To minimize potential confounds, behavioral tests were carried out between 07:30 AM to 1:30 PM and the testing order was randomized daily, with each animal tested at a different time on each testing day. All the stages of allocation, conduct of the experiment, outcome assessment, and data analysis were performed in a blinded manner. Statistical tests were performed using GraphPad Prism software (version 8.0, RRID: SCR_002798), and data were expressed as mean ± SEM. No outliers were identified in reported experiments, and no data were excluded from analyses except those from unsuccessful placements of intracranial injections. Statistical analysis was only undertaken for studies with each group size (n) ≥ 5. The statistical calculations performed by one-way ANOVA or two-way ANOVA were followed by Bonferroni's post hoc tests for multiple comparisons if F reached $P < 0.05$ and there was no significant departure from normal distribution and variance inhomogeneity. Two-tailed Student's *t*-test was used to compare the two groups. For all comparisons, differences were considered to be significant at $P < 0.05$.

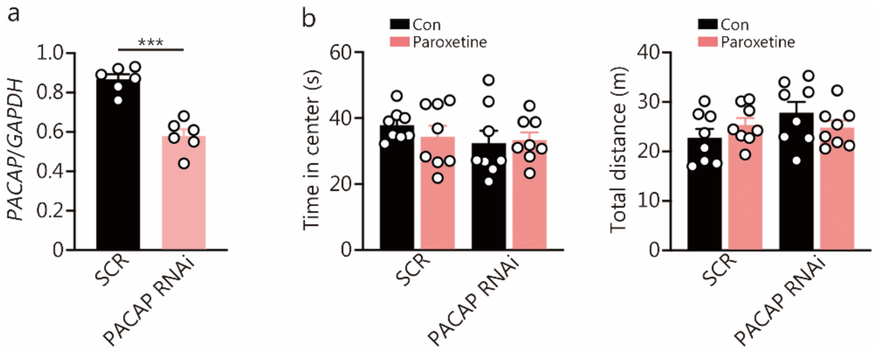

Fig. S1 Quantitative real-time polymerase chain reaction (PCR) analysis and open field test (OFT) after pituitary adenylate cyclase-activating polypeptide (PACAP) RNAi micro-infusion. **a** The *PACAP* expression was significantly reduced in the hippocampal *PACAP* knockdown RNAi mice ($n = 6$). *t*-test, $^{***}P < 0.001$. **b** Time in the center and total distance in OFT did not change in the hippocampal *PACAP* knockdown RNAi mice after paroxetine administration for 14 d [F (1, 28) = 0.5820, $P = 0.4519$; F (1, 28) = 2.623, $P = 0.1165$] ($n = 8$). Two-way ANOVA. Con control, SCR scramble control sequence

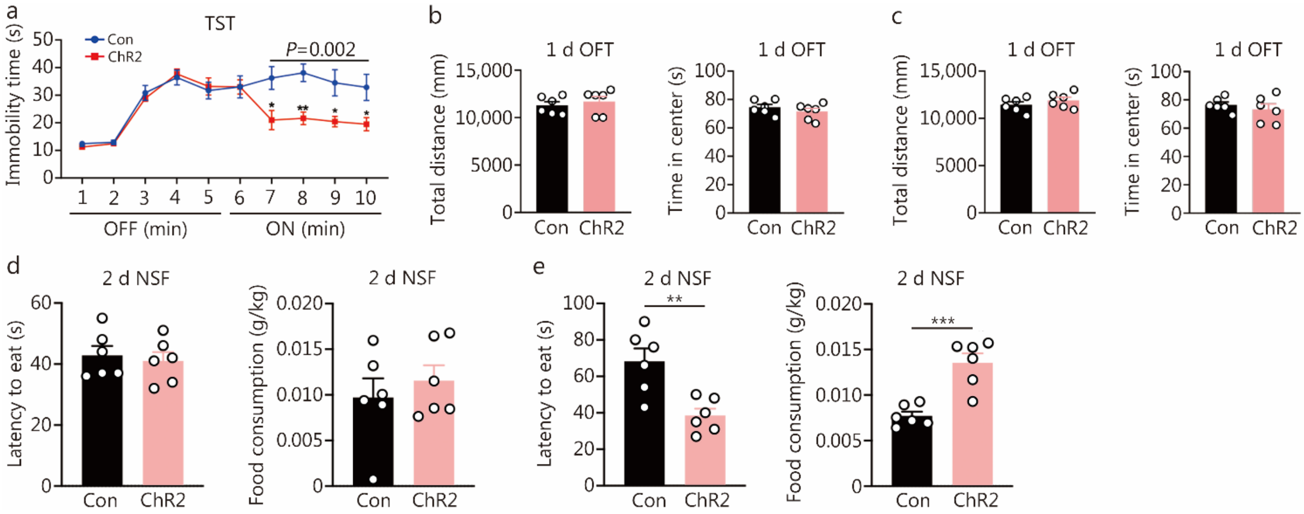

Fig. S2 Min-by-min analysis of the immobility time in the tail suspension test (TST) before and during optogenetic stimulation of pituitary adenylate cyclase-activating polypeptide (PACAP)-expressing neurons in the hippocampal dentate gyrus. **a** Immobility time for the initial 2 min and following 4 min in the absence of light, followed by another 4 min with light on for opto-stimulation. A min-by-min analysis indicated that there was no effect of time on the behavior, repeated *t*-test, time effect, $t = 1.46$, $P = 0.897$; optogenetic × time effect, $t = 10.11$, $P = 0.046$; 7 – 10 min, optogenetic effect, $t = 10.11$, $P < 0.001$. **b** Total distance and time in the center in the open field test (OFT) at 1 d post-opto-stimulation for 4 min ($n = 6$). *t*-test. **c** Total distance and time in center in OFT at 1 d post-opto-stimulation for 30 min ($n = 6$). *t*-test. **d** Latency to eat and food consumption of NSF test at day 2 post 4 min opto-stimulation ($n = 6$). **e** Latency to eat and food consumption of NSF test at day 2 post-30-minute opto-stimulation ($n = 6$). *t*-test, $^{**}P < 0.01$, $^{***}P < 0.001$. Con control, ChR2 channelrhodopsins 2

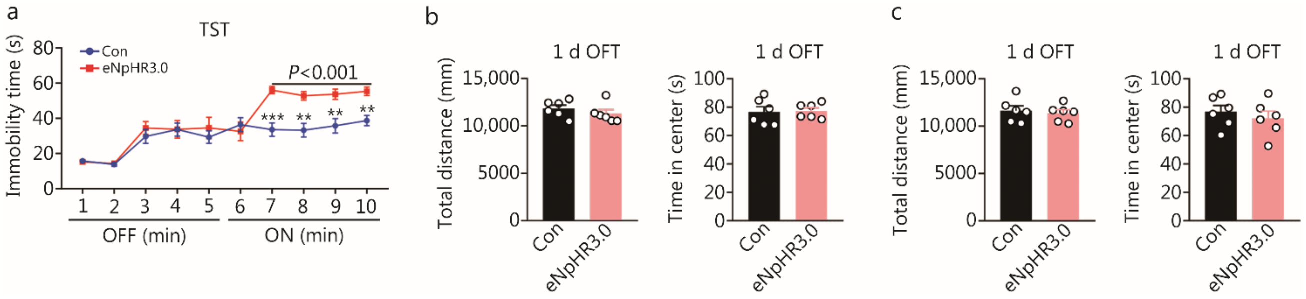

Fig. S3 Min-by-min analysis of the immobility time in the tail suspension test (TST) before and during optogenetic inhibition of pituitary adenylate cyclase-activating polypeptide (PACAP)-expressing neurons in the hippocampal dentate gyrus. **a** Immobility time for the initial 2 min and following 4 min in the absence of light, followed by another 4 min with light on for opto-inhibition $^{**}P < 0.01$ $^{***}P < 0.001$. A min-by-min analysis indicated that there was no effect of time on the behavior, repeated *t*-test, time effect, $t = 5.194$, $P = 0.966$; optogenetic × time effect, $t = 14.284$, $P = 0.004$; 7 – 10 min, optogenetic effect, $t = 14.441$, $P < 0.001$. **b** Total distance and time in the center in OFT at 1 d post-opto-inhibition for 4 min ($n = 6$). *t*-test. **c** Total distance and time in the center in OFT at 1 d post opto-inhibition for 30 min ($n = 6$). *t*-test. Con control, eNpHR3.0 endoplasmic natronomonas halorhodopsine 3.0

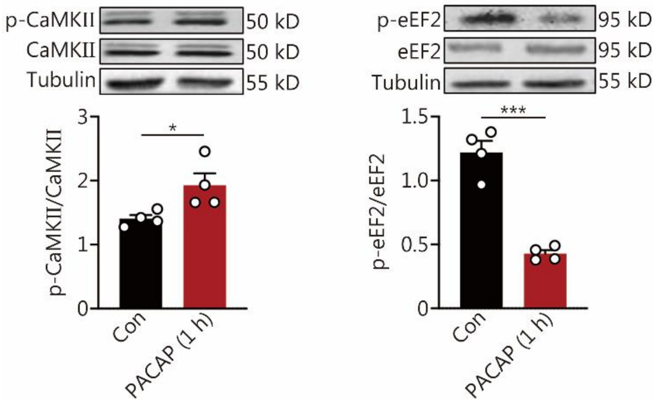

Fig. S4 Pituitary adenylate cyclase-activating polypeptide (PACAP) infusion regulated calcium/calmodulin-dependent protein kinase II (CaMKII)/eukaryotic elongation factor 2 (eEF2) signaling. Expressions of p-CaMKII/CaMKII and phosphorylated eukaryotic elongation factor 2 (p-eEF2)/eEF2 at 1 h following intra-hippocampal dentate gyrus PACAP infusion ($n = 4$). *t*-test, $^{*}P < 0.05$, $^{***}P < 0.001$. Con control

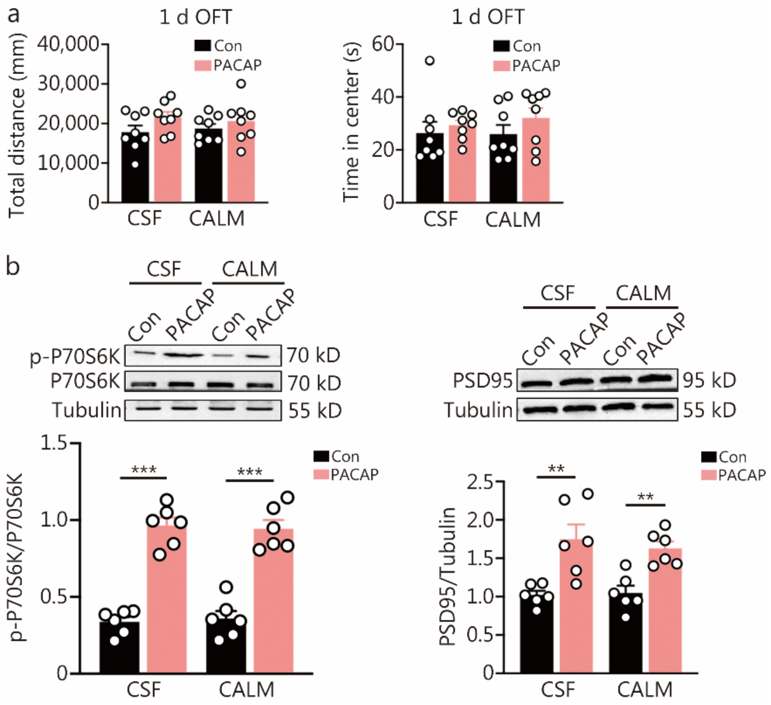

Fig. S5 The effects of intra-dentate gyrus (DG) microinfusion of calcium/calmodulin-dependent protein kinase II (CaMKII) agonist (CALM) on the immediate or lasting antidepressant activity of pituitary adenylate cyclase-activating polypeptide (PACAP). **a** Total distance and time in the central zone in the open field test (OFT) at 1 d post CALM and PACAP intra-DG microinfusion [F (1, 27) = 0.132, P = 0.719; F (1, 27) = 0.681, P = 0.415]. Two-way ANOVA. **b** The effects of preactivation of CaMKII on intra-hippocampal dentate gryus (HDG) pituitary adenylate cyclase-activating polypeptide (PACAP)-induced cell signaling responses. Expressions of p-P70S6K/P70S6K and PSD95 at 30 min after PACAP microinfusion. [F (1, 20) = 0.173, P = 0.681; F (1, 20) = 0.3639, P = 0.5531] (n = 6). Two-way ANOVA, $^{**}P < 0.001$, $^{***}P < 0.001$. CSF cerebrospinal fluid, PSD95 postsynaptic density protein-95, Con control

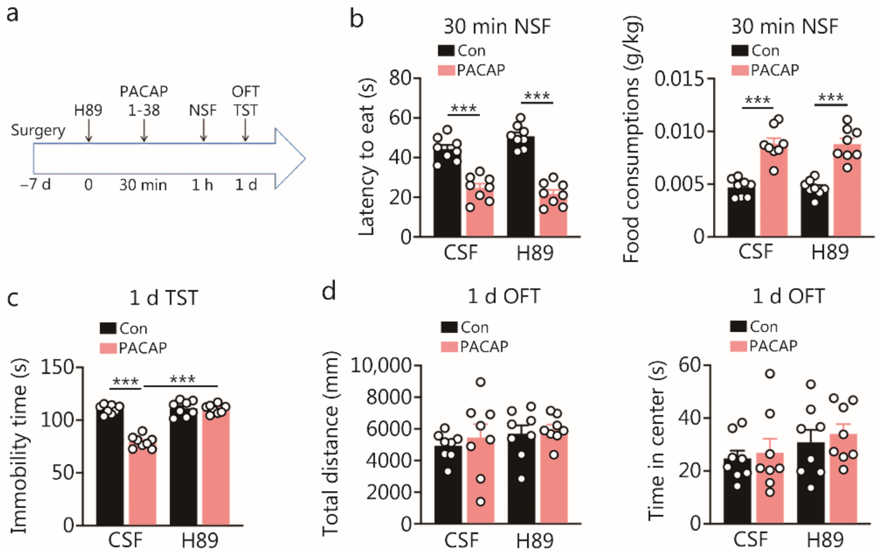

Fig. S6 Intra-HDG microinfusion of protein kinase A (PKA) antagonist H89 on the immediate or lasting antidepressant activity of PACAP. **a** The diagram for the experimental procedure of intra-HDG microinfusion of PKA antagonist H89 on the immediate or lasting antidepressant activity of PACAP. **b** Latency to eat and food consumption in the novelty suppressed feeding test (NSF) at 30 min post-H89 and PACAP microinfusion[F (1, 28) = 0.645, P = 0.429; F (1, 28) = 0.0003, P = 0.960]. Two-way ANOVA. **c** The immobility time in tail suspension test (TST) at 1 d post-hippocampal administration of PACAP pretreated with H89 [F (1, 28) = 60.61, P < 0.001]. Two-way ANOVA, $^{***}P < 0.001$. **d** Total distance and time in the central zone in the OFT at 1 d post-H89 microinfusion [F (1, 28) = 0.051, P = 0.824; time in the center: F (1, 28) = 0.014, P = 0.907] (n = 8). Two-way ANOVA. CSF cerebrospinal fluid, Con control

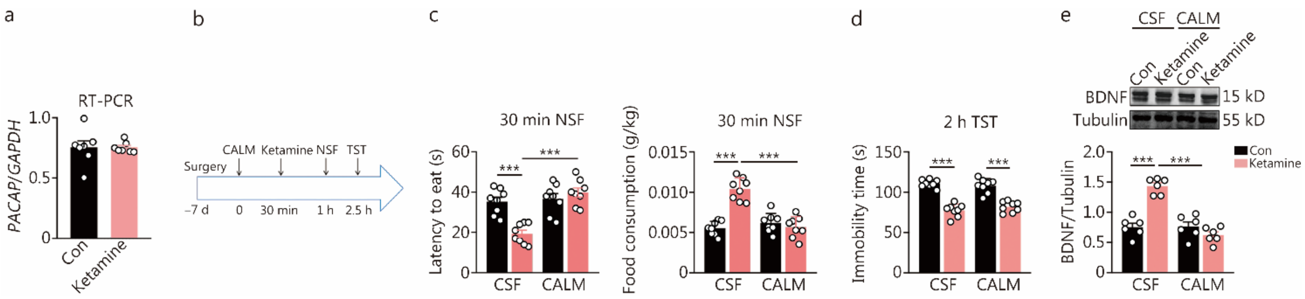

Fig. S7 The association of pituitary adenylate cyclase-activating polypeptide (PACAP) and related signaling with ketamine. **a** The *PACAP* expression in the hippocampus at 30 min post a single administration of ketamine (n = 6). t-test. **b** The experiment design for intra-dentate gyrus (DG) PACAP 6-38 microinfusion and ketamine. **c** Latency to eat and food consumption in the novelty suppressed feeding (NSF) at 30 min d post ketamine [F (1, 28) = 17.88, P = 0.0002; F (1, 28) = 39.84, P < 0.0001]. **d** Immobility time in the tail suspension test (TST) at 2 h post ketamine [F (1, 28) = 2.00, P = 0.1686]. **e** The expression of hippocampal brain-derived neurotrophic factor (BDNF) at 30 min post-intra-DG CALM microinfusion and ketamine [F (1, 20) = 0.173, P = 0.681; F (1, 20) = 43.13, P < 0.001] (n = 6 – 8). Two-way ANOVA, $^{***}P$ < 0.001. CSF cerebrospinal fluid, Con control

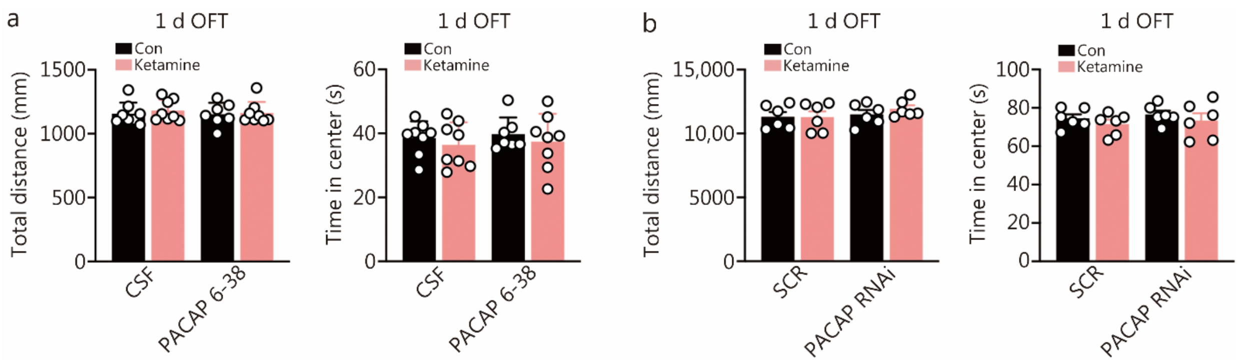

Fig. S8 Intra-dentate gyrus (DG) pituitary adenylate cyclase-activating polypeptide (PACAP) antagonism or *PACAP* knockdown treatment did not alter open field test (OFT) behavior along with ketamine administration. **a** Total distance and time in the central zone in the OFT at 1 d post intra-DG PACAP 6-38 microinfusion and ketamine [F (1, 27) = 0.064, P = 0.802; F (1, 27) = 0.002, P = 0.967] (n = 7 – 8). **b** Total distance and time in the central zone in the OFT at 1 d post intra-DG PACAP RNAi and ketamine [F (1, 20) = 0.372, P = 0.548; F (1, 20) = 0.001, P = 0.973] (n = 6). Two-way ANOVA. CSF cerebrospinal fluid, SCR scramble control sequence
